# Failure behavior of polymer microelectrode arrays encapsulated with conventional ALD and 3D-ALI barriers

**DOI:** 10.3389/fbioe.2025.1622927

**Published:** 2025-07-24

**Authors:** Martin Niemiec, Fatih Bayansal, Tejas Bhosale, Steven Suib, Necmi Biyikli, Kyungjin Kim

**Affiliations:** ^1^Department of Biomedical Engineering, University of Connecticut, Storrs, CT, United States; ^2^Department of Electrical and Computer Engineering, University of Connecticut, Storrs, CT, United States; ^3^Institute of Materials Science, University of Connecticut, Storrs, CT, United States; ^4^Department of Chemistry, University of Connecticut, Storrs, CT, United States; ^5^Department of Mechanical Engineering, University of Connecticut, Storrs, CT, United States

**Keywords:** atomic layer deposition, vapor phase infiltration, atomic layer infiltration, flexible bioelectrodes, lifetime analysis, failure mode analysis

## Abstract

As implantable electronics become thinner, softer, and more flexible, there is an increasing need for encapsulation strategies which enable these next-generation devices to survive for sufficient durations in the implanted environment. Atomic layer deposited (ALD) films of metal oxides have been studied for this purpose but suffer from intrinsic incompatibilities with soft and flexible substrates. Additionally, conventional fabrication processes often leave exposed sidewalls vulnerable to moisture permeation, undermining the effectiveness of the encapsulation. In this work, we report an encapsulation method based on atomic layer infiltration (ALI) which eliminates exposed sidewalls while remaining compatible with active microelectrodes for stimulation and recording. We compare the lifetime of sidewall-encapsulated (i.e., 3D) ALI devices under accelerated aging conditions to unencapsulated and conventional ALD-encapsulated groups. Overall, we find that while the 3D-ALI encapsulation successfully reduces sidewall vulnerabilities and offers qualitative improvements in degradation behavior compared to ALD, it did not significantly extend device lifespan. Taken together, these findings highlight both the promise of the 3D-ALI strategy and the need for further study and optimization.

## 1 Introduction

In recent years, the field of bioelectronics has shifted away from conventional implantable devices, which are often bulky and comprised of rigid materials, toward thinner, softer, and more flexible formats (Kathe et al., 2021; 2022; Michoud et al., 2021; Song et al., 2023), aiming for improved biointegration and long-term functionality (Cao et al., 2024; Kim et al., 2024; Boufidis et al., 2025). While these emerging devices have shown promising biocompatibility, they struggle to provide the mechanical and chemical stability required to withstand the harsh conditions of the implanted environment, limiting their broader adoption (Li et al., 2021; Mariello et al., 2022). This challenge is particularly critical for in-human applications, where device failure must be minimized to ensure patient safety and reduce the need for explantation and re-implantation procedures. Though significant advancements have been made in recent years toward commercial and clinical implementation of thin and flexible bioelectronic implants (Musk, 2019; Kullmann et al., 2022; Rapoport et al., 2024; Lee et al., 2025), more work remains to be done to ensure implants remain stable and functional over extended chronic timescales. Generally, lifetime enhancement of thin-film polymer implants has been focused on incorporating materials with superior water- and ion-barrier properties into neat polymers, such as atomic layer deposited (ALD) HfO_2_, Al_2_O_3_, TiO_2_, SiO_2_, and SiN_x_ (Minnikanti et al., 2014; Song et al., 2018; Jeong et al., 2019; Kim et al., 2021), as well as chemical vapor deposited (CVD) SiC (Frewin et al., 2011; Maboudian et al., 2013; Lei et al., 2016; Deku et al., 2018). Encapsulations incorporating these materials have been extensively tested *in vitro*, and have been shown to greatly extend lifetime when properly engineered. Critically, though, most *in vitro* studies of these advanced encapsulations assess the performance of the barrier material itself via purpose-built test devices such as interdigitated electrodes or permeation sensors. While these studies provide useful data on the degradation and failure behavior of the encapsulation itself, there is no guarantee that the encapsulation strategy can be effectively extended to the fabrication of therapeutically useful devices (*i.e.,* with exposed, active microelectrodes capable of recording and stimulation). For instance, the sequential microfabrication steps used to fabricate conventional planar microelectrode arrays involve depositing materials one layer at a time. This process can leave critical areas–such as device outlines and electrode via sidewalls–unprotected by the barrier material at the end of the fabrication process. These unprotected areas may then serve as initiation points for failure over time, reducing the effectiveness of the encapsulation (Ghelich et al., 2021). Because of these considerations, it is difficult for functional devices to achieve the nominal lifetime enhancement demonstrated by the encapsulation material *in vitro,* and the fabrication of functional devices incorporating these encapsulations is more challenging. Nonetheless, functional devices incorporating advanced encapsulation materials on flexible substrates have been produced, using fabrication techniques designed to mitigate at least some of these issues. For instance, Verplancke et al. (2019) developed a polyimide (PI)-based device with a polymer-ALD multilayer encapsulation. To avoid exposing the electrode via sidewalls, they created a stair-step structure in the multilayer stack and coated the entire surface with metal, effectively forming a large, continuous electrode without sidewalls. But even in such cases, as long sequential wafer-based fabrication is used, and the final ALD layer therefore deposited prior to the etching of device outlines, sidewall vulnerabilities will be created when the full stack is cut through to facilitate device singulation, meaning that even the most advanced encapsulation strategies stop short of completely eliminating vulnerable areas.

Permeation of bodily fluids into implanted microelectrodes can occur through any breach of the encapsulation layer that exposes the polymer substrate (Kim et al., 2021; Niemiec and Kim, 2023). The water vapor transmission rate (WVTR) through a polyimide substrate is highly sensitive to the size of exposed sites in the inorganic encapsulation layer, as demonstrated in studies of pinhole-based permeation. For example, the WVTR can vary by three orders of magnitude: from ∼8 × 10^−3^ g/m^2^/day for a 100 µm pinhole to ∼8 × 10^−6^ g/m^2^/day for a 100 nm pinhole, with 7 pinholes distributed across a 1 cm^2^ surface (Diyaroglu et al., 2023; 2024). This variation in WVTR has significant implications for long-term water absorption and, by extension, the integrity of the device. After 1 year, this variation in WVTR results in accumulated water sorption of 3 µm and 3 nm, respectively. In the lower WVTR case, the device could still maintain electrical functionality, even with submicron-thick metal track (Niemiec and Kim, 2023). While pinholes can be addressed by fine-tuning of encapsulation deposition parameters, the unprotected sidewalls on electrode openings and device substrate edges created when vias and outlines are etched can still introduce additional sites for water permeation. Advancing the sidewall encapsulation technique around electrode openings can enable complete sidewall encapsulation, effectively achieving seamless 3D encapsulation for active microelectrodes, but the question of how to accomplish this effect remains. One strategy is to create electrode vias, then deposit the ALD barrier on of them, followed by removal of the ALD material by etching. Although exposure of electrodes via ion beam milling of the overlying ALD layer has been demonstrated on-wafer (Westerhausen et al., 2020), it is not practicable on free, released devices because wafer processing techniques such as precise alignment can no longer be employed. Therefore, while the strategy can account for electrode via sidewalls, device outline sidewalls may still be unprotected if the last ALD layer is deposited on-wafer prior to outline etching.

Even if complete encapsulation of vulnerable areas by ALD barriers is achieved, the failure of encapsulation layers at interfaces with dissimilar underlying materials has long been recognized (Kim et al., 2021; Hsu et al., 2007). This issue is amplified when the substrate material is two orders of magnitude lower in elastic modulus compared to the encapsulation layer, thereby creating high elastic mismatch at the interface that significantly increases energy release rate, i.e., driving force for mechanical failures, under applied loading (Kim et al., 2016; 2017; 2018). Differently doped homogeneous all-SiC-based neural probes using crystalline and amorphous SiC for the recording site and encapsulation, respectively (Diaz-Botia et al., 2017; Bernardin et al., 2018; Beygi et al., 2019), may improve device reliability with seamless integration in the planar direction. However, the polymer-ceramic interface mismatch in the thickness direction is so far unresolved for polymer-based neural implants. Atomic layer infiltration (ALI) offers a promising approach to this challenge. By modifying ALD growth parameters (i.e., substrate temperature, ALD precursor doses, precursor diffusion (infiltration), purge cycles, and infiltration reactor pressure), ALD precursors can infiltrate the porous polymer matrix, forming a gradient modulus and therefore an ambiguous interface that resists interfacial delamination (Wilson et al., 2005; Lee et al., 2009; Peng et al., 2010; Akyildiz et al., 2012; Parsons et al., 2013; Z. Leng and D. Losego, 2017; Waldman et al., 2019; Petit et al., 2021).

**FIGURE 1:**
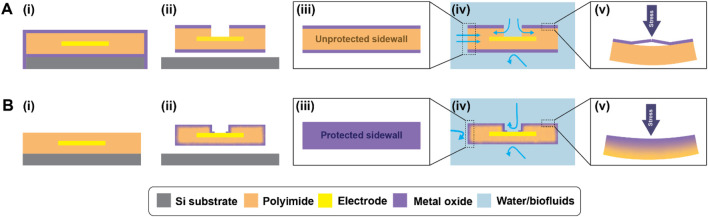
Comparison of **(A)** conventional ALD and **(B)** 3D-ALI encapsulation. (i) Cross section of on-wafer device prior to electrode via and device outline, etch, (ii) cross section of device after all fabrication and encapsulation steps are complete, (iii) outside view of finished device showing difference in sidewall protection of conventional encapsulation vs. 3D-ALI, (iv) device after submersion in fluid, with blue arrows highlighting possible paths for moisture and ion permeation showing reduced vulnerability of 3D-ALI coating, and (v) response of conventional ALD and 3D-ALI encapsulation to mechanical stress.

Here, we report a strategy for off-wafer 3D encapsulation of freestanding active microelectrode arrays, including electrode via and device substrate sidewall encapsulation, with the additional benefits conferred by an ALI hybrid structure, illustrated in Figure 1. The figure compares devices fabricated using conventional ALD methods (Figure 1A) with those fabricated using the 3D-ALI method (Figure 1B). The sidewall coverage enabled by 3D encapsulation of freestanding devices protects vulnerable areas from moisture permeation (Figure 1iv) and the ALI-derived hybrid material offers improved mechanical resilience when stressed (Figure 1v). In this work, we compare the electrochemical properties of 3D-ALI coated devices under simulated implanted conditions over time relative to unencapsulated and conventional ALD-encapsulated devices. We hope that this work further informs the feasibility of flexible, thin-film polymer-based bioelectronics as true long-term solutions for implanted applications.

## 2 Materials and methods

### 2.1 Polymer device fabrication

**FIGURE 2:**
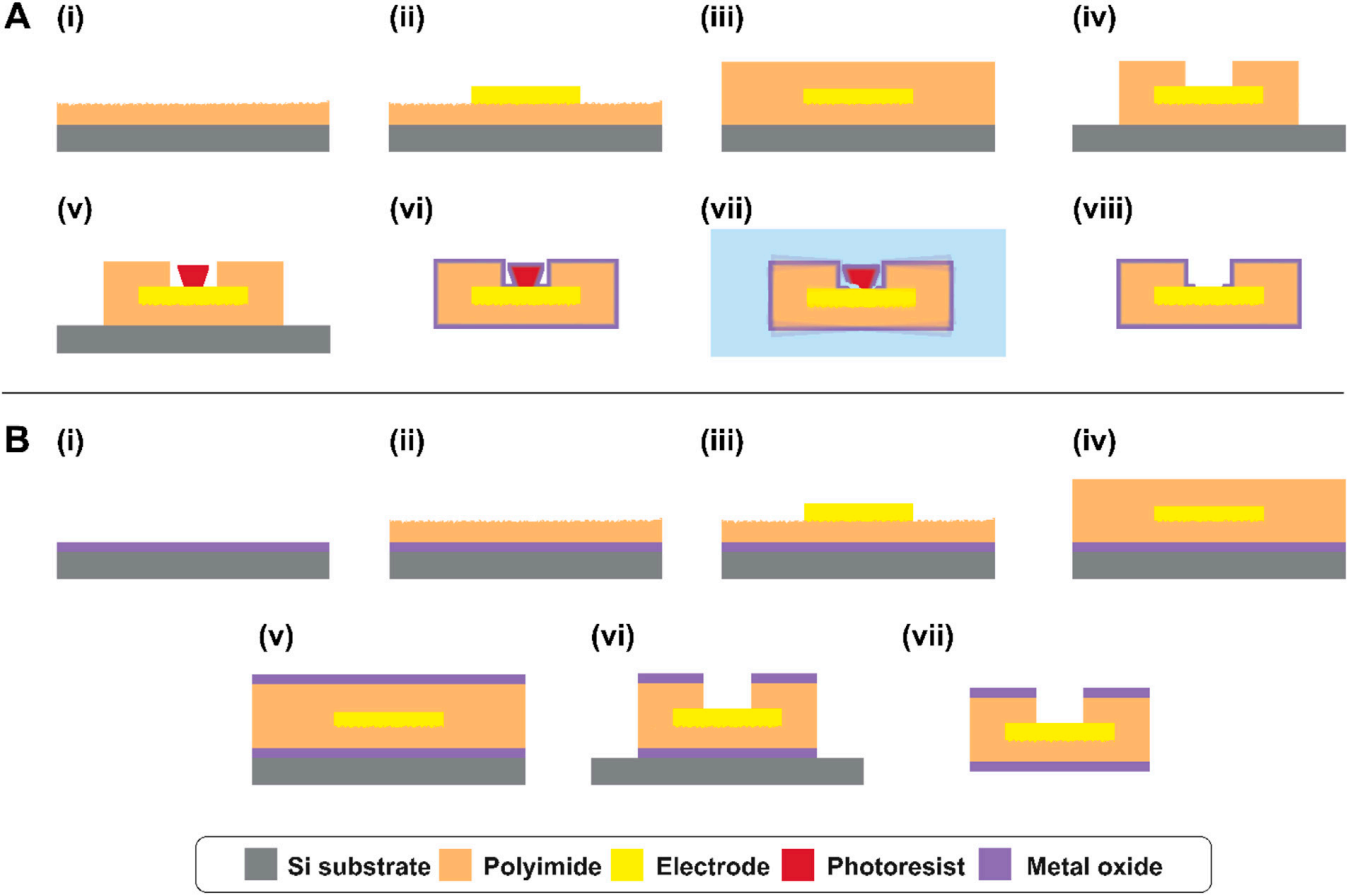
Device fabrication and via opening process for **(A)** ALI devices and **(B)** ALD devices. **(A)** (i) Bottom PI spincoating and O_2_ reactive ion etching (RIE) roughening, (ii) metal stack patterning, (iii) top PI spincoating and curing, (iv) outline and electrode via etching, (v) sacrificial layer lithography, (vi) whole-device encapsulation via atomic layer infiltration and deposition, (vii) removal of sacrificial resist via sonication in heated photoresist remover, and (viii) final device. **(B)** (i) Bottom ALD, (ii) bottom PI spincoating and O_2_ RIE roughening, (iii) metal stack patterning, (iv) top PI spincoating and curing, (v) top ALD, (vi) outline and electrode via etching, (vii) final device. Layers are not to scale.

Polymer devices were fabricated using standard microfabrication techniques. The thin-film microelectrode arrays consisted of a rectangular polyimide substrate of 2–3 µm thickness and 4.8 × 0.8 cm length and width, respectively, supporting five Pt microelectrodes with geometric surface area 1k-100 kµm^2^ depending on the device, encapsulated with another 2–3 µm polyimide and finished with a final ALD or 3D-ALI coating. The total number of devices tested was n_total_ = 17, with n_control_ = 4, n_ALD_ = 5, and n_ALI_ = 8. Fabrication was conducted in a clean room environment whenever possible to minimize contamination. First, a 4-inch silicon wafer (University Wafer, Boston, MA) was sonicated briefly in acetone, then rinsed thoroughly with isopropanol and deionized water and dried with nitrogen gas. 20 nm titanium and 100 nm magnesium were deposited using electron beam evaporation (ATC-2036 HV Series, AJA International, Hingham, MA) to serve as a sacrificial layer facilitating device release at the end of fabrication. Then, PI2610 polyimide precursor (HD Microsystems, Parlin, NJ) was spin-coated onto the wafer at 1500RPM for 30 s. The PI2610 was softbaked on a hot plate at 130°C for 3 min, then cured at 205°C for roughly 5 h. After curing, the base layer of PI2610 was roughened using reactive ion etching in oxygen plasma at 50W RF power, 5mTorr chamber pressure, and 40sccm feed rate for 45 s (Vision 320 MK II, Advanced Vacuum/Plasma-Therm, St. Petersburg, FL) (Figure 2i). Next, AZ5214E-IR photoresist (Merck Performance Materials GmbH, Darmstadt, Germany) was spincoated onto the surface at 2000RPM for 40 s and softbaked for 2 min and 20 s at 100°C. The resist was patterned for metal liftoff using a mask aligner (Hybralign 200, OAI, Milpitas, CA) and in-house fabricated photomasks. A first exposure of 0.3 s was followed by a reversal bake of 2 min and 20 s at 113°C, then 20 s no-mask flood exposure, and development in 130 mL AZ400K 1:3 photoresist developer (Merck Performance Materials GmbH, Darmstadt, Germany) and 70 mL water with agitation until undercut was visible, about 50 s. The resist was then hardbaked for 10 min at 100°C to ensure stability during metal deposition. A metal stack consisting of 30 nm chromium, 20 nm platinum, 100 nm gold, 20 nm platinum, and 20 nm chromium was deposited using electron beam evaporation (Figure 2ii). 20 min cooling was allowed after platinum steps to prevent excessive heating. Chromium was used for top and bottom adhesion, and platinum was used to separate gold and chromium to limit alloy formation during subsequent high-temperature curing steps, also serving as the microelectrode surface. Following deposition, excess metal was removed by liftoff in room-temperature acetone. The second layer of PI2610 was spincoated at 2000RPM for 30 s and softbaked as before. However, the top layer’s cure temperature was increased to 300°C to ensure full curing of the top and bottom layers together (Figure 2iii) and conducted under a nitrogen atmosphere. For electrode via and device outline etching, thick photoresist (AZ P4620, Merck Performance Materials GmbH, Darmstadt, Germany) was patterned as an, etch mask. Spincoating at 2000RPM for 60 s was followed by softbaking at 117°C with gradual reductions in height prior to full contact to avoid bubbling of the resist. After softbaking, the resist was allowed to rest on a warm, damp wipe in a sample container for at least 1 h to facilitate rehydration. The resist was then exposed using the same mask aligner in two 3-s intervals, spaced slightly to reduce heating and avoid nitrogen evolution during exposure, and developed in undiluted AZ400K 1:3 with agitation for about 5 min. As a hardbake step, the softbake was repeated plus an additional 3 min of full contact. Electrode vias and device outlines were etched using 300W oxygen plasma at 300mTorr for about 8 min, until the electrodes were exposed but the bottom polyimide was not completely etched (Figure 2iv). Chromium etchant (standard, Sigma-Aldrich, St. Louis, MO) diluted 50:50 with water was used at room temperature with agitation to remove top chromium from the electrode surface and expose the platinum beneath, about 35 s. At this point, the resist was removed using room-temperature acetone or AZ Kwik Strip resist remover at 50°C–60°C (Merck Performance Materials GmbH, Darmstadt, Germany) and re-patterned in the same fashion. This was done to prevent lateral over-etching of the electrode via during the second phase of plasma exposure. The remaining polyimide was etched using the same plasma recipe for an additional 8–9 min. Remaining resist was removed using acetone or Kwik Strip. AZ5214E-IR photoresist was then patterned in image reversal mode as before (but with no or minimal hard-bake) on the electrode sites, to serve as a sacrificial layer for ALD via opening (Figure 2v). At this point, microfabrication is complete. 0.125 mm Teflon-coated silver wires (World Precision Instruments, Sarasota, FL) were soldered to the bond pads using a low-temperature solder (SMDSWLTLFP32, Chip Quik Inc., Ancaster, ON, Canada), and sealed with silicone (DOWSIL 732, Dow Inc., Midland, MI). The entire wafer was then immersed in room temperature saline solution with stirring for about 2 h, until the sacrificial magnesium was dissolved and the devices were gently released from the wafer.

For ALD-integrated devices, the same overall fabrication steps were followed, with the key difference that ALD layers were deposited before and after the polyimide steps while the devices remained on the wafer (Figure 2Bi,v and (v)). These layers were etched using SF_6_ RIE and HF-free titanium etchant (Multi, Etch, USA) before performing oxygen plasma etching of the polyimide (Figure 2Bvi). All other fabrication steps were kept consistent with the standard process.

### 2.2 Atomic layer infiltration (ALI) and deposition (ALD)

**FIGURE 3:**
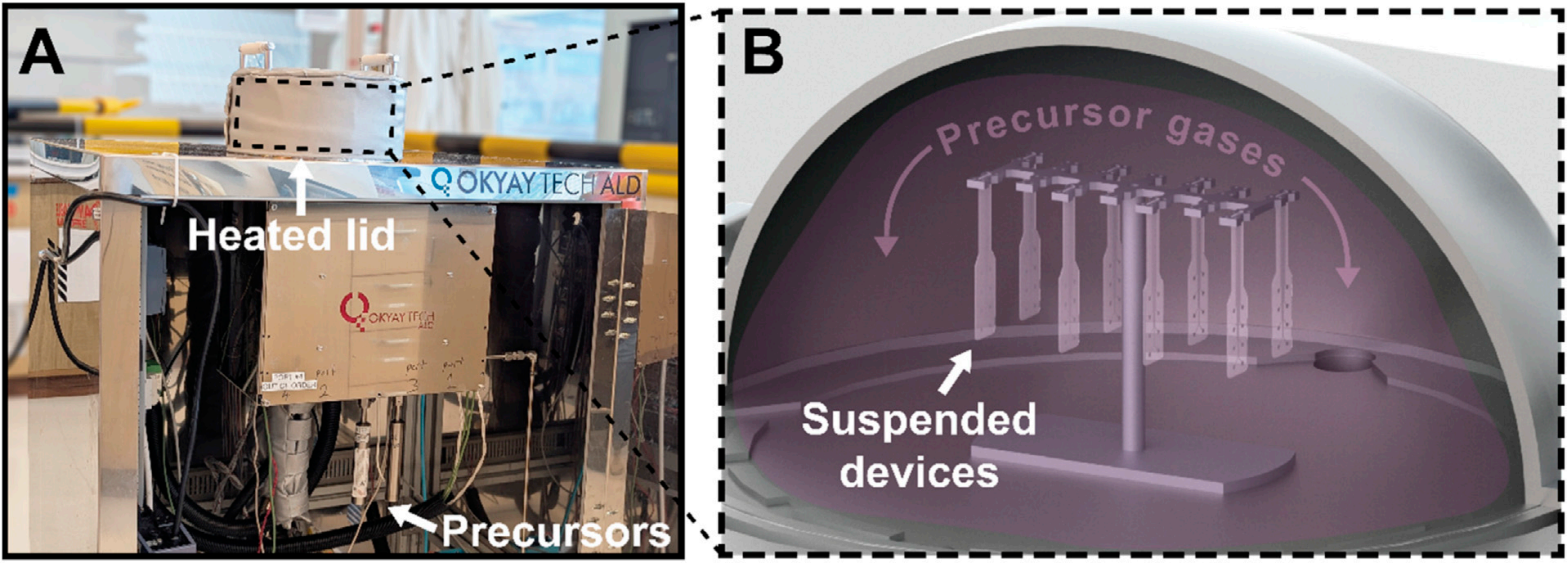
Illustration of 3D-ALI setup. **(A)** Photograph of thermal ALD system with custom heated domed lid used for deposition **(B)** Schematic illustrating 3D-ALI concept, wherein devices are suspended above the substrate and thereby exposed to precursor gases on all surfaces during deposition.

For ALI devices, once the devices were released from the wafer and the integrity of the sacrificial photoresist verified using an optical microscope, the devices were completely encapsulated on all sides with a bilayer of metal oxides (Figure 2Avi). This was done by suspending the devices vertically inside a domed-lid ALD chamber (Figure 3A), represented schematically in Figure 3B. By hanging the devices vertically instead of resting them on the chamber substrate, precursor molecules can reach active sites over the entire device uniformly, resulting in whole-device encapsulation. The device’s top and bottom surfaces are coated with the barrier material, as well as the electrode vias and sidewalls created by plasma etching, leaving no vulnerable polymer uncoated. The hybrid barrier consisted of aluminum oxide infiltrated into the polymer to create a gradient from pure polymer to pure metal oxide, capped with a layer of titanium dioxide serving to isolate the alumina from fluids. ALI and ALD were conducted in a thermal ALD chamber with a custom domed lid (Okyaytech ALD, Ankara, Turkey).

For ALI, trimethylaluminum (TMA) (Strem Chemicals, Newburyport, MA) and water vapor were used as precursors, targeting a chamber pressure of 6 Torr to encourage infiltration (Table 1). First, the chamber was pumped to base pressure and the gate valve closed. Then, TMA was pulsed repeatedly until the desired pressure was reached using the lowest possible carrier gas flow rate (5sccm N2). Once the desired pressure was reached, carrier flow was shut off, and the TMA vapor was allowed to dwell inside the reactor for 3 min (the “diffusion time”). A relatively short diffusion time of 3 min was chosen to facilitate multiple pulsing along the lines of sequential infiltration synthesis (“SIS”), as this approach may be better suited for gradient creation rather than the more uniform loading resulting from long single-cycle processes (Ham et al., 2022). After the diffusion time, the chamber was purged by alternating cycles of 100sccm carrier flow and pumping down to base pressure each for 1 min for a total of 6 min purging. More thorough purging results in a more ALD-like process within the substrate than a short purge, which can “trap” unbound precursor remaining inside the substrate by reacting with it during the next step (Ham et al., 2022). Following this, water vapor was pulsed, allowed to diffuse, and purged along the same parameters. The ALI/SIS process was repeated a total of 60 times, intended to be more than enough to fully saturate the free volume of the polymer near the surface, resulting in a film which is essentially ALD on top of the hybrid gradient. This was done to ensure that a uniform layer of water vapor diffusion-blocking alumina was present throughout the surface. After ALI, traditional thermal ALD titania was deposited using Tetrakis (dimethylamino) titanium (IV) (TDMAT) heated to 75°C (Strem Chemicals, Newburyport, MA) (100 ms) and water vapor (30 ms) with constant carrier flow 20sccm, targeting a thickness of ∼30 nm. For conventional ALD devices, two bilayers of 525 cycles titania (deposited as above) and 60 cycles alumina deposited using TMA (20 ms) and water vapor (30 ms) with carrier flow 20 sccm were deposited on-wafer, prior to electrode via/device outline etching and subsequent device release. All ALD/ALI processes were performed at 120°C. After deposition, film thicknesses were checked on reference Si wafer pieces using a multi-wavelength ellipsometer (Film Sense, Lincoln, NE).

**TABLE 1:** Summary and comparison of atomic layer infiltration (ALI) and deposition (ALD) parameters. When alumina ALD was performed, pulse durations were 30 ms and 20 ms for TMA and H2O, respectively.

	ALI	ALD
Precursors	Trimethylaluminum, H_2_O	Tetrakis (dimethylamino)titanium, H_2_O
Temperature	120°C	120°C
Diffusion time	3 min	N/A
Targeted pressure	6 Torr	N/A
Purge time	6 min	30 s
No. cycles	60	525
Precursor heating	N/A	TDMAT 75°C

### 2.3 Encapsulation via opening

Self-aligned encapsulation via opening was achieved by a combination of on- and off-wafer processes. Because the sacrificial layer is deposited while the devices are on the wafer, precise alignment of the sacrificial photoresist cap and electrodes is possible; then, because the encapsulation and subsequent via opening are performed off-wafer on freestanding devices, device outlines are etched before the last ALD layer is deposited, and therefore all surfaces including sidewalls are covered at the end of the process. Of course, after 3D encapsulation, the devices are conformally coated, including the electrode sites intended to be exposed; therefore, a modified liftoff strategy was employed to remove the deposited oxides from the electrodes while leaving encapsulation intact in the desired areas. First, the devices were mounted in the glass vials later used for accelerated aging tests by mounting their wires through the cap using a two-part epoxy (EA-30CL, Henkel Corporation, Stamford, CT). The vials were then partially filled with AZ Kwik Strip photoresist remover and placed into a water bath heated to 60°C and allowed to equilibrate thermally. Then, individual vials were transferred to a sonication bath (Air Control, Inc., Henderson, NC) and exposed to 72 kHz ultrasound at 25%–30% power for 30 s (Figure 2vii). After sonication, each vial was returned to the water bath to re-equilibrate with occasional mild agitation while the other vials were treated. Status of ALI/ALD removal was monitored using an optical microscope, noting changes in appearance and color as well as using focus variation to determine whether the oxide-coated resist was still present on the electrode. The cycle was repeated as necessary. Once ALI/ALD removal was determined to be complete for all sites, the devices were thoroughly rinsed with isopropanol and deionized water. At this point, the devices were considered clean and ready for study.

### 2.4 Film characterization

To better understand the properties of the ALD/ALI encapsulated polymer film, various analysis techniques were employed. Scanning electron microscopy (SEM) with energy dispersive X-ray spectroscopy (EDS) (Teneo LVSEM, Thermo Fisher Scientific, Waltham, MA) was used to map the distribution of elements on the surface and thereby confirm removal and retention of the ALI/ALD coating from electrode surfaces and on device sidewalls, respectively. Secondary Ion Mass Spectrometry (SIMS) measurements were performed using a Hiden Analytical Compact SIMS. The instrument was connected to an ion interference unit, a MAXIM quadrupole mass spectrometer, and an electron interface unit. Secondary ion mass spectra, ion mapping, and depth profiles were recorded using Ar + as primary ions with an average current of 10 mA and energy of 5keV, using a sputter size of 2000 μm × 2000 μm, an analysis area of 1,000 µm in diameter, and a 1 µm deep crater. The water vapor transmission rate (WVTR) of a similar film was measured using an Aquatran 3 permeation analyzer (MOCON, Inc., Brooklyn Park, MN) at 37°C and 50% relative humidity (RH), in order to demonstrate the capability of the hybrid encapsulation in reducing permeation of water vapor.

### 2.5 Accelerated aging tests

Finally, the effectiveness of the encapsulation at prolonging the lifespan of the devices under simulated implanted conditions was assessed using accelerated aging tests wherein the devices were maintained in phosphate buffered saline (PBS) at 87°C. Based on Baker’s rule, which is derived from the Arrhenius equation, an increase in temperature can be correlated to an increase in chemical reaction rates, and therefore used to simulate aging for longer periods of time (Hukins et al., 2008). Generally, an increase of 10°C is considered to double the rate of relevant chemical reactions, and therefore double the “aging” a device experiences in the same amount of time. This can be described mathematically as *a* = 2^ΔT/10^ where *a* is the acceleration factor and ΔT is the temperature difference. At a testing temperature of 87°C, the accelerated aging factor relative to 37°C is *a* = 2^(87–37)/10^ = 32, *i.e.*, every day spent at 87°C is roughly equivalent to 32 days spent at 37°C. While useful, it is important to note that the doubling relationship as described has been shown to become less accurate above certain temperatures (∼70°C) (Li et al., 2020), and therefore the lifetimes reported from accelerated aging should be taken as rough estimates. However, because all devices studied experienced the same accelerated aging factor, differences between control and experimental groups can still inform their longevity relative to each other, which is of primary interest here.

Four key data were collected daily for the duration of the experiment: electrochemical impedance and phase at 1 kHz, determined from electrochemical impedance spectroscopy (EIS); charge stored per phase, determined from cyclic voltammetry (CV); and optical images of electrode surfaces. EIS spectra were collected from 0.1 Hz to 100 kHz at an amplitude of 50 mV, and CV was conducted by sweeping potential between −0.6V and +0.8V a total of three times to ensure stability at a sweep rate of 100 mV/s, with only the final CV cycle used for analysis. In both cases, an Ag/AgCl reference and platinum wire counter electrode were used in PBS maintained at 37°C in a heated water bath (Corning, Inc., Corning, NY) with the microelectrode as working electrode. Data were collected using a CHI 660E electrochemical workstation (CH Instruments, Bee Cave, TX). Optical images were acquired using a Nikon Labophot 2 microscope (Nikon Corporation, Tokyo, Japan) and Amscope software (AmScope, Irvine, CA). The devices were tested electrochemically, then immersed briefly in deionized water to remove residual PBS solution, dried, and gently placed under a clean glass microscopy slide or otherwise held flat for consistent imaging before being returned to their vials and placed in an oven maintained at 87°C.

### 2.6 Data analysis

EIS and CV data were imported, processed, and visualized using a custom MATLAB script. Charge per phase was calculated by integrating CV current vs. time. Box plots were generated in RStudio using stat_compare_means to test for significant differences via Wilcoxon rank-sum test. Weibull analysis was performed using Python, specifically the Fit_Weibull_2P function from the Reliability library.

## 3 Results

### 3.1 Fabrication improvements enabling ultrasonication-assisted via opening

After fabricating test devices using previously established methods, it was observed that adhesion between the devices’ component layers (illustrated in supplementary data, Supplementary Figure S1A) was not sufficiently robust for combination with ultrasonication-assisted via opening. Although the oxide layer over the electrodes was successfully removed during sonication, delamination of the top polyimide from the metal traces was visible (Supplementary Figure S1B, left), as well as damage to the electrodes themselves (Supplementary Figure S1C, top), making the devices unusable for accelerated life testing. To address these issues, fabrication parameters were modified to improve interlayer adhesion. Based on previous studies on partial curing (Ceyssens and Puers, 2015) and plasma-based substrate roughening (Cen-Puc et al., 2021), the base polyimide layer was partially cured at 205°C (rather than the standard 300°C) and treated with reactive ion etching oxygen plasma prior to subsequent layer deposition to improve polyimide-polyimide adhesion. In addition, given the known poor adhesion between polyimide and gold (Cen-Puc et al., 2021), an additional metal layer was introduced to the metal stack to promote polyimide-top metal adhesion. Chromium was chosen, as it could be easily removed using a liquid chromium etchant during fabrication in order to expose the electrode surface. Platinum was also added as the electrolyte-contacting material, with gold still forming the bulk of the trace for improved conductivity. Devices fabricated with the revised metal stack and additional adhesion-promoting treatments showed no trace delamination (Supplementary Figure S1B, right) and electrodes remained intact (Supplementary Figure S1C, bottom) after sonication. With these modifications, the encapsulation via opening process and overall device structure were deemed optimized and ready for further characterization.

### 3.2 Ultrasonication-assisted via opening and comparison with “conventional” ALD

**FIGURE 4:**
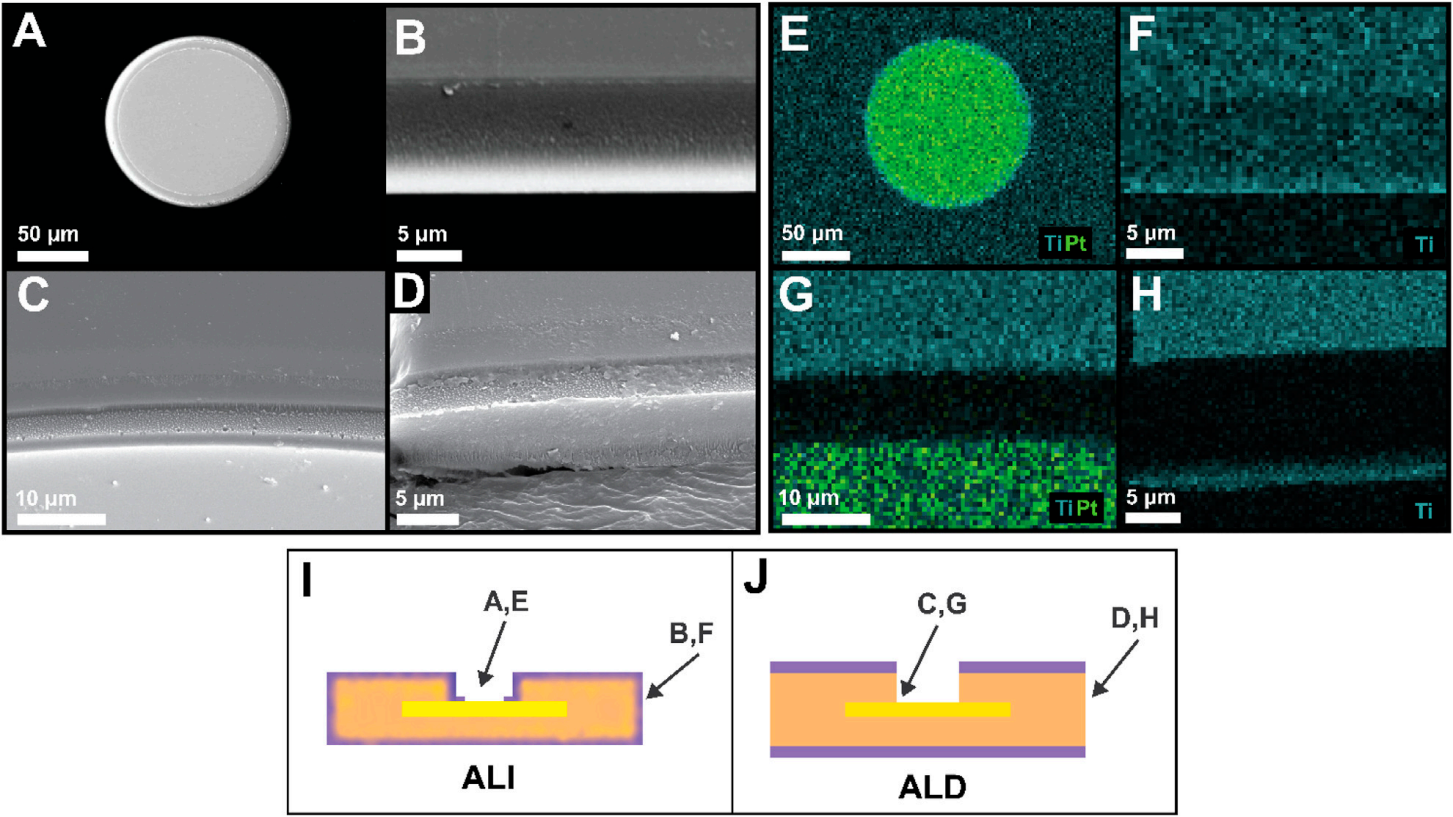
Verification of electrode via opening and sidewall encapsulation. **(A)** Scanning electron microscope (SEM) image of an electrode post-ALI/ALD via opening **(B)** SEM image of an ALI device’s outer sidewall from a 45-degree angle. The light-colored region on the bottom edge of the device was most likely due to charging resulting from directionality of AuPd sputtering prior to imaging. **(C)** SEM detail of an ALD device electrode (light gray), electrode via sidewall, and ALD-coated PI (darker gray), taken from a 45-degree angle **(D)** SEM of an ALD device’s outer sidewall from a 45-degree angle **(E)** EDS elemental map of the electrode depicted in A showing no titanium (light blue) present on electrode surface beyond a small edge ring, but presence elsewhere throughout **(F)** EDS map of ALI device sidewall shown in B, demonstrating retention of titania on device sidewall after encapsulation via opening **(G)** EDS map of area shown in C, showing that titania is present on the top surface but an unprotected sidewall has been created (dark area) during electrode via opening **(H)** EDS map of area shown in D, again showing an unprotected sidewall created by device outline etching. In G and H, unprotected (dark) areas are slightly larger than the sidewalls themselves due to over-etching during fabrication. For additional clarity, **(I,J)** indicate the field of view of panels A–H as they relate to device structure.

While the 3D-ALI strategy was designed to accomplish encapsulation and protection of device and electrode via sidewalls while simultaneously leaving electrodes exposed, it was necessary to confirm that both of these aims were achieved. Initial validation was performed during fabrication, during which oxide-coated photoresist was visible in the center of the electrode as a reddish circle (Supplementary Figure S2A) but no longer present after ultrasonication treatment in heated photoresist remover (Supplementary Figure S2B). Further validation was achieved via SEM-EDS: SEM images of the electrode surface confirmed that the electrode surface was flat and the photoresist cap was not present (Figure 4A), and EDS elemental mapping showed that titanium (blue) and therefore titania was not present on the platinum surface (green) barring a thin ring where there was no photoresist prior to deposition (Figure 4E). The width of this ring could be reduced by increasing the diameter of the photoresist cap, but doing so would make photomask alignment more challenging. Figure 4B shows a device’s outer sidewall, with EDS confirming that titania remained on the sidewall after it was removed from the electrode (Figure 4F), accomplishing the combination of sidewall protection and electrode via opening targeted by our 3D-ALI method. In contrast, Figures 4C,D,G,H show similar regions on a device encapsulated by “conventional” top and bottom ALD. Figure 4C shows a closeup of an ALD device electrode and its via sidewall from a 45° angle, with Figure 4G showing the distribution of titanium (blue) and platinum (green). The dark area shows the polymer sidewall unprotected by any ALD material. Similarly, Figure 4D shows an ALD device’s sidewall, with titanium visible in blue on the top and bottom of the device but not on the sidewall (Figure 4H). Figures 4I,J schematically illustrate the viewing perspectives of panels A-H to contextualize what part of the device is depicted in each. These images illustrate the vulnerable areas left behind by the conventional ALD approach and demonstrate that our 3D-ALI approach helps address this issue.

### 3.3 ALD and ALI encapsulation film properties

**FIGURE 5:**
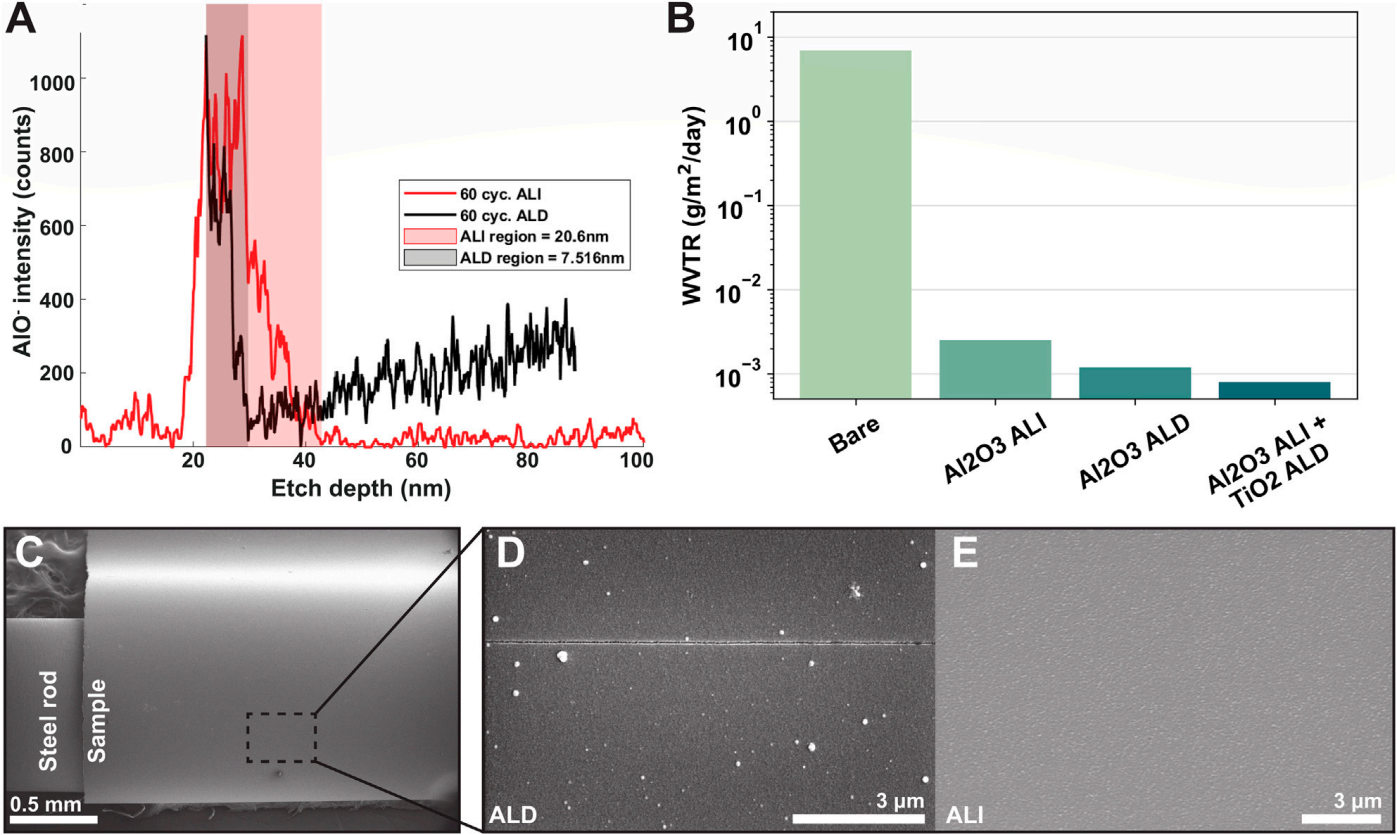
ALD and ALI film properties. **(A)** SIMS profile of aluminum content vs. depth for an infiltrated (red) and ALD (black) sample. Shaded area indicates approximate aluminum-containing region for each sample, with the infiltrated sample showing a greater width. The increase in aluminum signal following the initial decrease in the ALD sample is likely an artifact of the SIMS technique. Etch depths are approximate. **(B)** Water vapor transmission rate comparison of various encapsulations on 5 mil Kapton HN samples **(C)** Critical radius of curvature testing setup. ALD **(D)** and ALI **(E)** samples were curved around a rod of known radius and observed for cracking via SEM. At r = 0.5 mm, no cracking was visible on the ALI sample, while cracks were observed on the ALD sample.

Next, we characterized the properties of the deposited hybrid encapsulation. Per ellipsometry on reference Si wafer pieces included in the deposition, expected alumina/titania layer thicknesses were about 10.5/34.5 nm for ALD devices, and 16.3/34 nm for ALI. Layer thicknesses on the devices themselves may vary slightly based on the presence and extent of infiltration or the nucleation behavior of alumina on silicon vs. polyimide, though the difference is quite small on most polymers (Wilson et al., 2005). Figure 5A shows a SIMS depth profile of alumina content, specifically AlO^−^, for an infiltrated sample (red) and an ALD sample (black). AlO^−^ signal is depicted by a line of the appropriate color, and the “alumina-containing region” (*i.e*.*,* the region from alumina signal onset to offset) is shaded in the same color. When the surfaces of the alumina-containing regions are aligned, it can be seen that the alumina-containing region of the ALI sample (red) is wider than the alumina-containing region of the ALD sample (black). The, etch depth reported on the x-axis is an approximated value, as it could not be measured directly and is difficult to estimate for hybrid materials whose compositions (and therefore, etch rates) change with depth. Nonetheless, the SIMS depth profiles provided a preliminary indication that infiltration was achieved as desired. Further verification was attempted using transmission electron microscopy (TEM) of cross-sections with EDS; however, localized observations in the analyzed regions did not reveal the same extent of infiltration suggested by the SIMS results. This may reflect nonuniform infiltration depth across the sample surface, as supported by later findings. Alternatively, even limited infiltration of a few nm may have been sufficient to account for the observed material properties.

Figure 5B shows the WVTR data of ALI and ALD encapsulations applied to a 5-mil Kapton HN polyimide substrate. At 50% relative humidity and 37°C, bare polyimide had a WVTR of 6.95 g/m^2^/day. When coated with 100 cycles of alumina ALI, the WVTR decreased to 2.5 × 10^−3^ g/m^2^/day; when coated with 100 cycles alumina ALD, the WVTR was 1.2 × 10^−3^ g/m^2^/day. Finally, when coated with a combination of 100 cycles alumina ALI +250 cycles titania ALD, the WVTR was 0.8 × 10^−3^ g/m^2^/day, approaching the detection limit of the system (0.5 × 10^−3^ g/m^2^/day). An example of a full WVTR test curve is shown in the supplementary data (Supplementary Figure S3). While WVTR does not directly correlate with lifetime, because many corrosion reactions involve water, it is generally beneficial to reduce the amount of water which permeates through the encapsulation over a given period as much as possible. With the ALI/ALD encapsulation, the WVTR was reduced to about 0.8 × 10^−3^ g/m^2^/day at 50% RH, or a barrier improvement factor of more than three orders of magnitude, confirming that our hybrid encapsulation serves as a good barrier to water vapor. Even at an increased humidity of 85%RH, the WVTR was nearly the same at 0.9 × 10^−3^ g/m^2^/day.

Next, to assess whether the encapsulation exhibited improved mechanical properties, we performed a rough estimation of its critical radius of curvature by bending coated samples around rods of known radii and examining the presence or absence of cracks using SEM (Figure 5C). Cracks were observed on the ALD-coated sample at a bending radius of 0.5 mm (Figure 5D), corresponding to a surface strain of approximately 0.8%, based on the device thickness of 8 μm. In contrast, no cracks were observed on the ALI-coated sample at the same bending radius (Figure 5E). Ellipsometry data indicated a total ALI thickness of 50.3 nm (comprising 16.3 nm of alumina and 34 nm of titania) and a total ALD thickness of 45 nm (10.5 nm alumina and 34.5 nm titania). Despite the ALI film being similarly thick—or even slightly thicker—and composed of the same brittle materials, its crack onset strain (COS) was higher than that of the ALD film. This is attributed to the alumina portion in the ALI film being infiltrated, which alters its mechanical behavior. Thus, the observed COS trend does not follow the expected inverse relationship with thickness (Jen et al., 2011; Kim et al., 2019). Cracks in the ALI-coated sample were finally observed at a smaller bending radius of 0.254 mm, suggesting a COS between 0.8% and 1.6%.

### 3.4 Device failure modes

**FIGURE 6:**
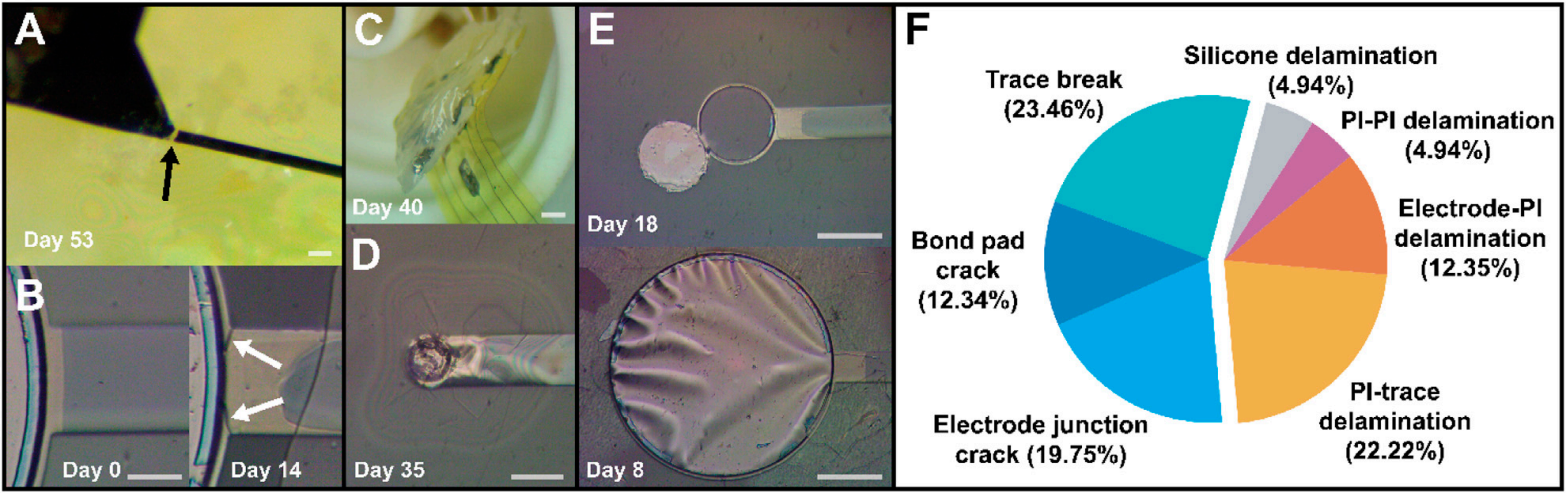
Various failure modes exhibited by devices during accelerated aging. **(A)** metal cracking near the bond pad and **(B)** at the electrode-trace junction. **(C)** Delamination of silicone near the bond pad area and **(D)** delamination between upper and lower polyimide layers (PI-PI), as well as between top polyimide and top metal (PI-trace). **(E)** Adhesion failure between bottom metal and polyimide (Electrode-PI) resulting in complete detachment (upper image) and “billowing” (lower image) of the electrode surface. **(F)** A pie chart showing prevalence of individual failure modes among sites at the point of total failure, with metal cracking related failures in the left segment and adhesion-related failures in the right segment. Scale bars are 50 µm, 25 µm, 2 mm, 50 µm, and 100 µm, respectively.

Before providing a statistical treatment of the failure data, it is important to understand the various modes in which electrode sites experienced failure during aging. Failures could be categorized into roughly seven unique phenomena, depicted and summarized in Figure 6. Broadly speaking, failures stratified into two groups: cracks and delamination (adhesion failure). Cracking was primarily observed in the metal stack, which resulted in increased impedance and decreased charge storage due to the effective open circuit and reduction in electrode surface area. Metal cracks formed most often in three areas: either at the interface between the wire bond pad and the trace itself (Figure 6A), at the junction between the electrode and the trace (Figure 6B), or on the bond pad itself. This type of failure occurred with greater frequency than expected, with more than half of sites experiencing one of these varieties of metal cracking by the time of total failure (Figure 6F, left segment). The high prevalence of this failure mode was unexpected because the devices were not intentionally mechanically stressed in any way; therefore, it was not predicted there would be significant drivers of crack formation. The cause of these cracks was then somewhat unclear, but may have been driven by thermal cycling as devices were heated to 87°C during aging, cooled to room temperature prior to testing, and maintained at 37°C during electrochemical analyses. Cracking at the bond pad itself is theorized to result from thermal stresses during the electron beam evaporation step, due to the high power required to evaporate platinum resulting in significant heating. This was reduced prior to final device fabrication by adding a gold interlayer to the metal stack in order to reduce total platinum thickness, as gold requires much less power to deposit (∼16% vs. ∼40%). However, the persistence of occasional bond pad cracking suggests that platinum thickness (20 nm) should be further minimized, and additional cooling periods between metal deposition steps or perhaps substrate cooling during metal deposition implemented if possible. Metal cracking was particularly undesirable, as it occurred with similar frequency across all encapsulation groups, making it more difficult to isolate the encapsulation’s impact on device lifespan. An analysis of lifetimes excluding metal crack-related failure was attempted, but the reduction in sample size made analysis impractical. Cracking at the electrode junction primarily affected large electrodes, as the sudden increase in the trace width was a source of stress concentration.

Beyond metal cracking, device failure was primarily driven by adhesion loss, resulting in interlayer delamination. In a few cases, delamination occurred between the silicone protecting the bond pad area and the underlying PI substrate (Figure 6C). However, adhesion failure was more commonly observed between PI and top metal (“PI-trace”), or between top and bottom PI (“PI-PI), both visible in Figure 6D. Premature loss of adhesion between PI and bottom metal was seen in some cases (Figure 6E), most likely due to over-etching of adhesion-promoting chromium during the top chromium removal/platinum exposure step in liquid chromium etchant. This could be avoided by utilizing a dry, etch such as an SF_6_ RIE process or similar (Hoang Nguyen et al., 2021). Like electrode junction cracking, large sites were the most affected by this issue, as the “undercut” area was larger, sometimes even visible as “billowing” of the electrode as seen in Figure 6E (bottom image). Small sites affected by this issue were less impacted, as the PI-PI adhesion appeared sufficiently strong to hold them in place despite the loss of adhesion to the underlying layer. The “best” sites (*i.e.,* ones which were not affected by failures stemming from process imperfections) tended to fail by PI-trace or PI-PI failures, as even when the metal remained perfectly intact, the layers would eventually separate, resulting in decreased impedance and unreasonably large charge storage. Corrosion did not seem to be a major source of failure in these devices, potentially due to the bulk of the metals used being noble metals such as platinum and gold. Corrosion and dissolution of the adhesion-promoting chromium were occasionally visible (for example, see the change in chromium (gray) area between days 0 and 14 in Figure 6B), which may have contributed to loss of PI-trace adhesion, but PI-trace delamination was also observed in many cases where the top chromium appeared to be intact, suggesting that adhesion becomes insufficient even when significant corrosion and dissolution do not occur. When chromium was intact on the top and bottom, bottom metal-PI adhesion appeared less likely to fail than PI-top metal adhesion, most likely due to the oxygen RIE treatment of the base polyimide layer. An adhesion promoter such as aminopropyltriethoxysilane (APTES) on the top chromium could help reduce this discrepancy (Kuliasha and Judy, 2021).

**FIGURE 7:**
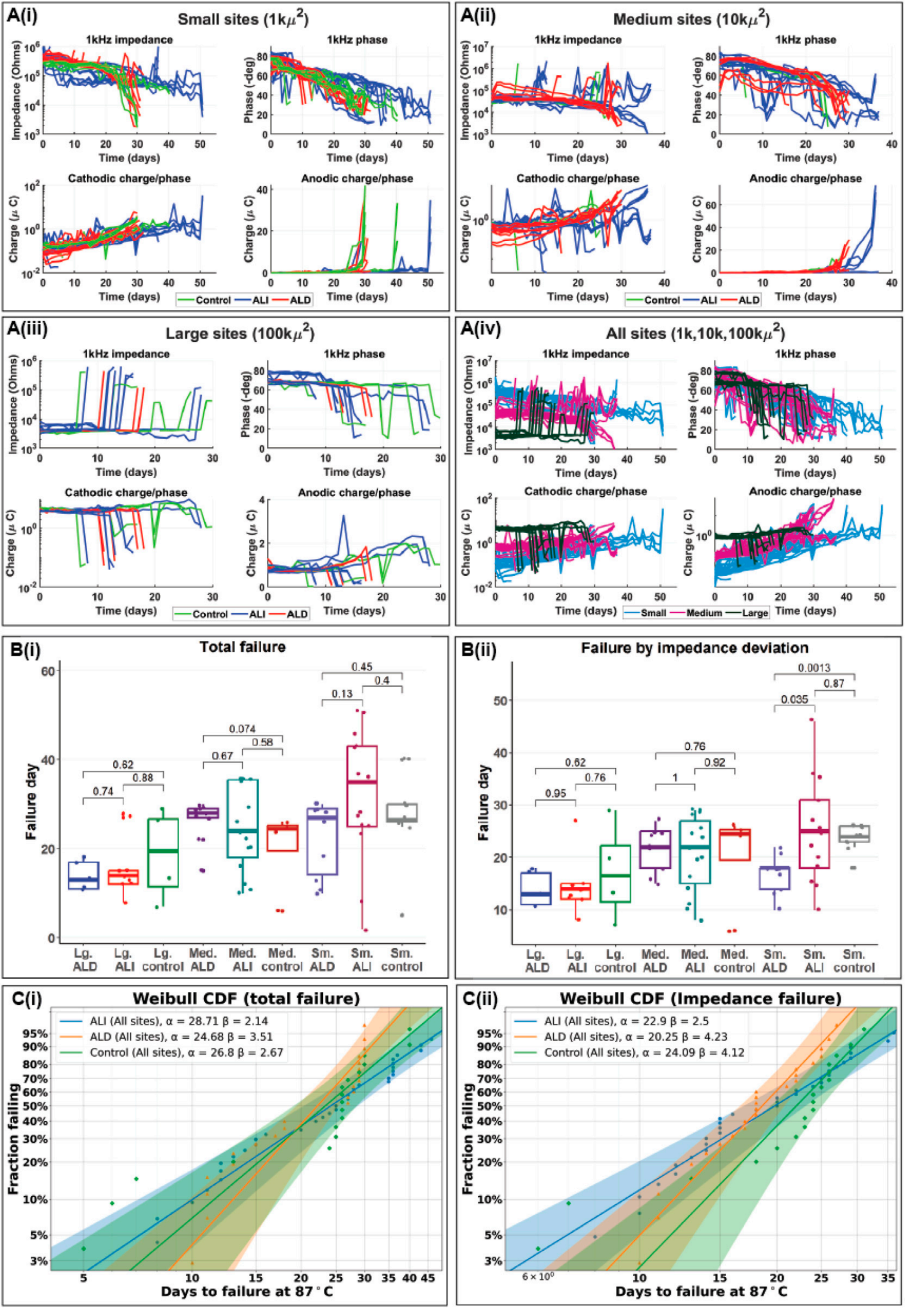
Results of accelerated aging tests in phosphate buffered saline at 87°C. **(A)** impedance and phase at 1 kHz derived from electrochemical impedance spectroscopy and cathodic and anodic charge-per-phase derived from cyclic voltammetry over time for small (1kµm^2^) (i), medium (10 kµm^2^) (ii) and large (100 kµm^2^) (iii) electrodes as well as all three sizes at once (iv). In (i-iii), a linear scale is used for anodic charge to highlight sudden increases in charge stored as electrodes approached total failure; charge storage is otherwise depicted using a log scale. **(B)** Box plots showing mean time to failure of electrodes with different surface areas and coatings when failure is evaluated by time to total failure (i) and deviation of more than (+/−) 50% from stable impedance setpoint (ii) **(C)** Weibull probability plots/cumulative distribution functions (CDFs) for electrodes of all sizes with different coatings when failure is defined by total failure (i) and impedance deviation (ii).

After devices were fabricated and encapsulated, we evaluated whether the improved encapsulation strategy resulted in tangible improvements to device lifespan under accelerated aging conditions. Because very long lifetimes were targeted, accelerated aging tests were performed in phosphate buffered saline at 87°C to ensure a high acceleration factor, nominally 32 times faster aging than at 37°C. However, as noted in the Methods section, the actual acceleration factor may have been lower. Three coating groups were compared: uncoated control devices, “conventional” top- and bottom-only ALD, and our 3D-ALI, hereafter referred to as “control”, “ALD”, and “ALI”. Three different electrode geometries were also studied for each group, with geometric surface areas (GSAs) of 1,000 μm^2^, 10,000 μm^2^, and 100,000 μm^2^, as electrodes of different sizes may experience failure differently. For example, platinum dissolution is known to occur at different rates based on the charge density present on the electrode surface (Shepherd et al., 2019), which will vary with the size of the electrode. These groups are hereafter referred to as “small”, “medium”, and “large” sites, respectively. Average day 0 impedances at 1 kHz for small ALI, small ALD, and small control electrodes were 9.93 × 10^5^, 3.65 × 10^5^, and 2.89 × 10^5^ Ω. For medium sites, the values were 5.78 × 10^4^, 4.49 × 10^4^, and 2.56 × 10^4^ Ω; for large sites, 6.67 × 10^3^, 3.98 × 10^3^, and 3.20 × 10^3^ Ω. Impedance values for ALI sites were generally higher than the other groups as a result of the ALD ring remaining around the outside of the electrode surface after sacrificial photoresist removal. Figure 7 shows three different ways of summarizing the results of the accelerated aging tests. A(i)-A (iv) show traces of the actual data collected over time to illustrate trends, and how they differ between treatment groups. Clockwise from top left are shown impedance at 1 kHz, phase at 1 kHz, cathodic charge-per-phase, and anodic charge-per phase, collected from EIS and CV, respectively. Though it does not capture a full picture of an electrode’s failure state, impedance at 1 kHz is often used as a metric for the health of electrodes intended for neural recording, as it is a useful range for detection of neural spiking (Han et al., 2024). Cathodic and anodic charge-per-phase (µC) were used instead of charge storage capacity (CSC, µC/cm^2^), as CSC is normalized to electrode surface area, which we found to be variable over time in certain failure modes. For example, in cases of PI-trace delamination, the effective electrode surface area increases, and assuming a constant area would lead to inaccurate charge density calculations. Figure 7Ai depicts trends for small (1,000 μm^2^) sites, with the control group shown in green, ALI in blue, and ALD in red. A (ii) and A (iii) depict the same for medium and large sites, respectively, while A (iv) compares sites on the basis of size irrespective of coating, with small sites in blue, medium sites in pink, and large sites in dark green. Figure 7Bi describes failure in terms of “total failure”; that is to say, when the site has been determined to have become completely non-functional based on changes in electrochemical characteristics as well as inspection visually and under optical microscopy. All sites and devices were tested until complete failure to ensure there was no ambiguity regarding the maximal extent of device lifetime, which can be unclear when experiments are stopped after a predetermined amount of time rather than after failure. This metric is somewhat subjective, as it relies on the operator’s assessment of the cumulative evidence of failure available for a given site, but it can sometimes provide a more complete picture of an electrode’s failure state than relying on a single metric. For example, if multiple failure modes are present, their effects can counteract each other in ways that are difficult to distinguish via a single metric, such as in the case of progressive corrosion (increasing impedance) coinciding with delaminating encapsulation (decreasing impedance). These failure modes together may result in a small net change of impedance, which would suggest a healthy electrode, while optical inspection might reveal significant deterioration. Nonetheless, an alternative analysis is presented in B(ii), wherein failure is strictly defined as a variation in 1 kHz impedance by more than (+/−) 50% from the site’s initial stabilized value, such as in Gong et al., 2020. Figure 7Ci and C(ii) present Weibull cumulative distribution functions (CDFs) representing total device failure and failure due to impedance variation, respectively, where the time is shown on the x-axis, and the fraction of devices having failed by that time is shown on the y-axis. Parameters for each distribution are provided in the legend, where α is the scale parameter and β is the shape parameter of the Weibull distribution. The three coating groups (ALI, ALD, and control in blue, orange, and green respectively) are compared in each plot, based on data from all three electrode sizes. The shaded areas represent 95% confidence intervals for each fit. Individual combinations (*e.g.,* small sites | with ALD) were also analyzed in this way (Supplementary data, Supplementary Figure S4).

Barring sudden changes arising from metal crack failures, most sites experienced a gradual decrease in 1 kHz impedance and phase along with a corresponding gradual increase in charge storage, consistent with a gradual increase in electrode surface area. Generally, survival appeared to be correlated with electrode size, with small electrodes surviving the longest and large electrodes the shortest. This is most likely due to an increasing propensity for cracking at the electrode junction with electrode size, as metal cracks developed more rapidly than other failure modes. When metal crack failures were excluded, the difference in total failure between groups based on size became nonsignificant (Supplementary data, Supplementary Figure S5). Many sites which failed by decreasing impedance began to show a strong peak in the anodic region of the CV curve which was not present at the start of testing. Because platinum does not have a strong anodic peak in the potential range tested, this could suggest that platinum dissolution had occurred, exposing the gold underneath, as the peak is consistent with gold oxidation (O’Neill et al., 2004). When analyzing by total failure, statistically significant differences did not exist between the failure distributions of the ALI, ALD, or control groups for a given electrode size. When failure was defined by impedance variation, small sites with ALD appeared to perform worse than those with ALI or even the control group. However, given the lack of a clear distinction between the ALI and control groups, encapsulation type did not appear to have a strong influence on device lifespan under the conditions tested. Weibull distributions were consistent with experimental observations, as all groups exhibited shape parameters β > 1, indicative of wear-out failure modes. The β value for the ALI group was generally lower than that of the ALD group, while the scale parameter α was higher. This reflects the qualitative observation that ALI devices began to show signs of failure earlier than ALD devices, but their failure progression occurred more gradually. These findings suggest that, with mitigation of early-stage failures, ALI encapsulation may enhance the long-term lifespan of devices. Mean times-to-failure derived from the Weibull distributions are given in Table 2 below, as well as equivalent lifetimes at 37°C assuming worst- and best-case acceleration factors of 8x (no additional acceleration past 67°C) and 32x, the nominal value. Again, no significant differences were found except between small ALD/control and small ALD/ALI when defining failure by impedance variation. While mean 37°C equivalent lifetimes ranged from about 0.5 to 2.75 years depending on the acceleration factor used, a few outlier sites survived until 51 days or 1.12/4.47 years, giving some indication that MTTFs may be improved with further optimization.

**TABLE 2:** Mean times to failure in days at 87°C derived from Weibull analysis when considering total failure (left) and failure by impedance variation (right). Sample size (n) refers to the total number of electrode sites in a given category across all devices and is slightly smaller when analyzing by impedance because some sites technically did not fail by this metric (test was stopped for other reason). 37°C equivalent lifetimes are given assuming no additional acceleration past 67°C (*a* = 8) and acceleration given by Baker’s rule (*a* = 32).

	Total Failure				Impedance change ±50%			
Group	MTTF (days)	Lifetime at 37°C with *a* = 8	Lifetime at 37°C with *a* = 32	n	MTTF (days)	Lifetime at 37°C with *a* = 8	Lifetime at 37°C with *a* = 32	n
ALI (all sizes)	25.42	0.56	2.23	39	20.78	0.46	1.82	37
ALD (all sizes)	22	0.49	1.95	24	18.07	0.40	1.58	23
Control (all sizes)	24.08	0.53	2.11	19	21.3	0.47	1.87	17
Small ALI	31.45	0.69	2.76	15	25.13	0.55	2.20	13
Small ALD	22.53	0.49	1.97	10	16.82	0.37	1.47	9
Small Control	27.23	0.60	2.39	10	23.62	0.52	2.07	9
Medium ALI	24.7	0.54	2.16	15	20.82	0.46	1.82	15
Medium ALD	26.42	0.58	2.31	9	21.64	0.47	1.90	9
Medium Control	21.91	0.48	1.92	5	20.19	0.44	1.77	4
Large ALI	16.05	0.35	1.41	9	14.4	0.32	1.26	9
Large ALD	14.03	0.31	1.23	5	14.02	0.31	1.23	5
Large Control	18.82	0.41	1.65	4	17.32	0.38	1.52	4

**FIGURE 8:**
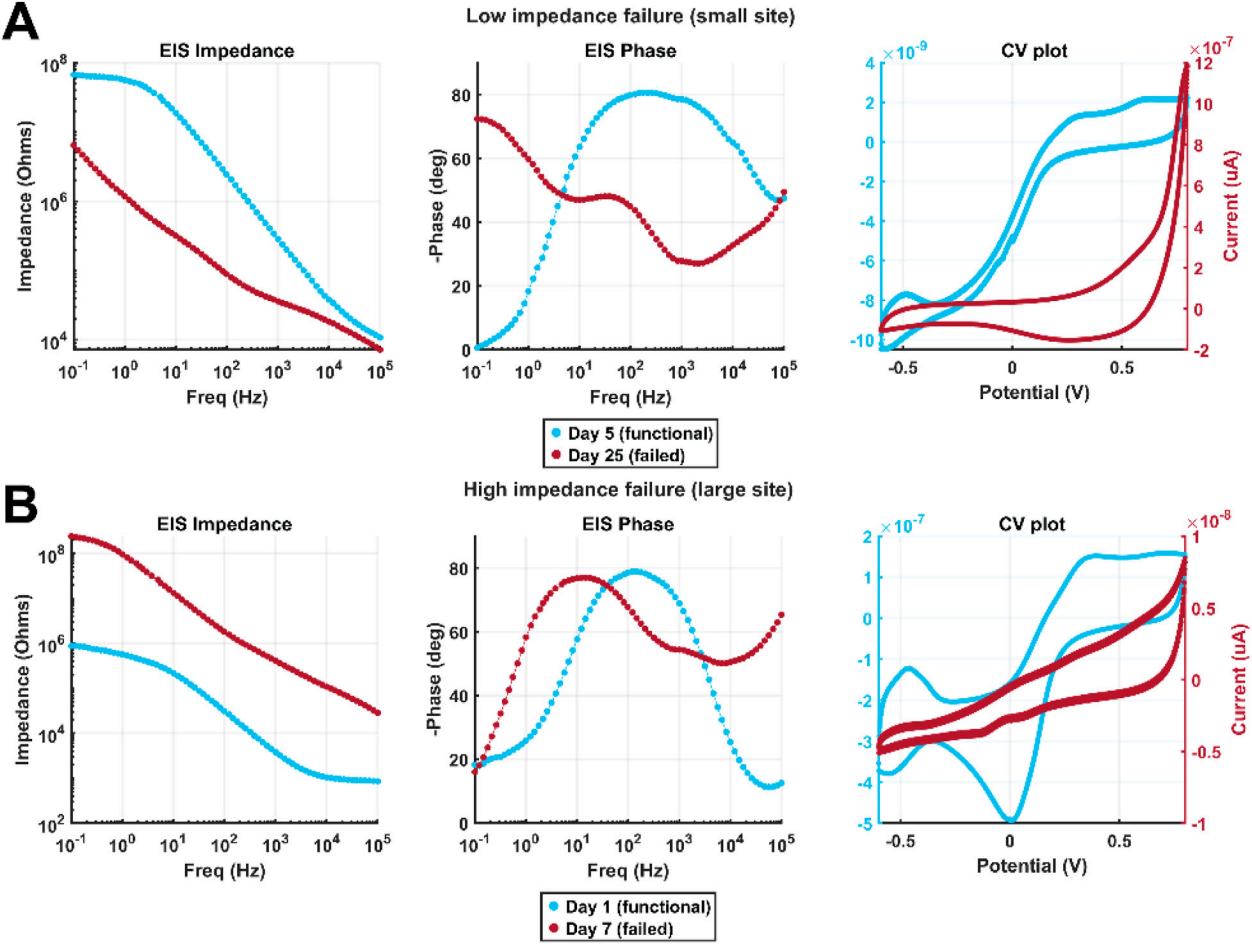
Example EIS spectra and CV plots comparing functional and failed sites in cases of **(A)** low-impedance failure and **(B)** high-impedance failure. Light blue and red traces are from the same electrode on a day which the electrode was functioning normally and one after it had failed, respectively. The data shown in A come from a small electrode which failed by PI-trace delamination, and B from a large electrode which failed by metal cracking at the electrode junction.

Figure 8 shows example data illustrating characteristic low-impedance and high-impedance failure. Data collected from the site prior to failure are shown in light blue, and data collected after failure are shown in red. Figure 8A illustrates behavior typical of low-impedance failure caused by PI-trace delamination, evident from the decrease in impedance at all frequencies as well as a transition to a more “hockey stick”-shaped impedance spectrum more similar to that of a large site, decrease in 1 kHz phase, and increase in charge storage as well as development of an anodic peak possibly suggestive of platinum dissolution. Figure 8B illustrates behavior typical of a metal crack at the electrode junction, evidenced by increased impedance and decreased charge storage. When optical microscopy did not render the cause of failure immediately clear, these patterns were useful in assigning a cause of failure for a given site.

### 3.5 Encapsulation failure

**FIGURE 9:**
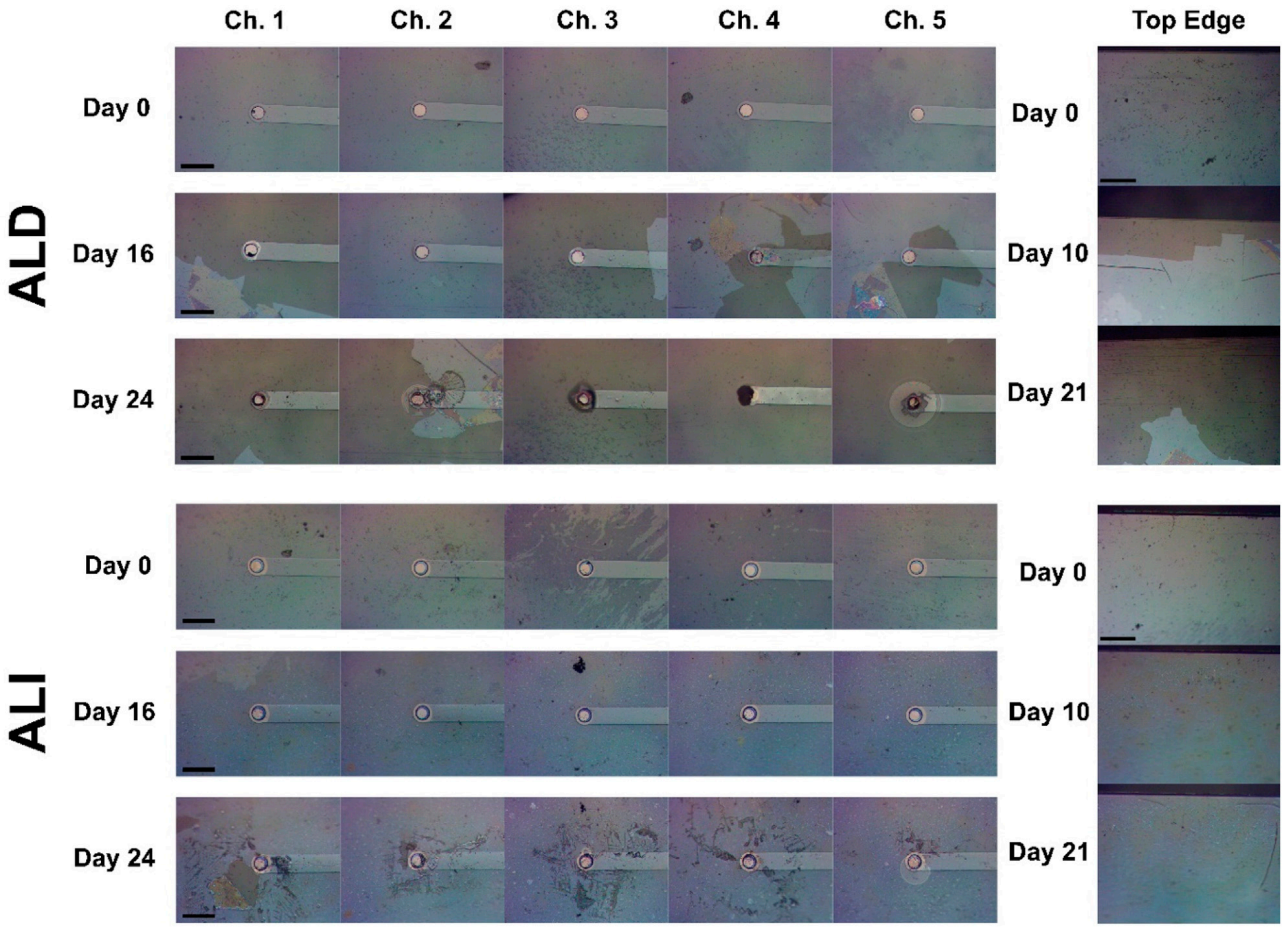
Progression of metal oxide coating degradation in the vicinity of electrode openings and device edges for ALD devices (top) and ALI devices (bottom). Left images show each of a device’s 5 electrodes at 3 different time points, illustrating the progression of coating degradation near the electrode. Right images show the edge of the device at 3 different time points, illustrating the progression of coating degradation near the device sidewall. Scale bar 100 μm.

**FIGURE 10:**
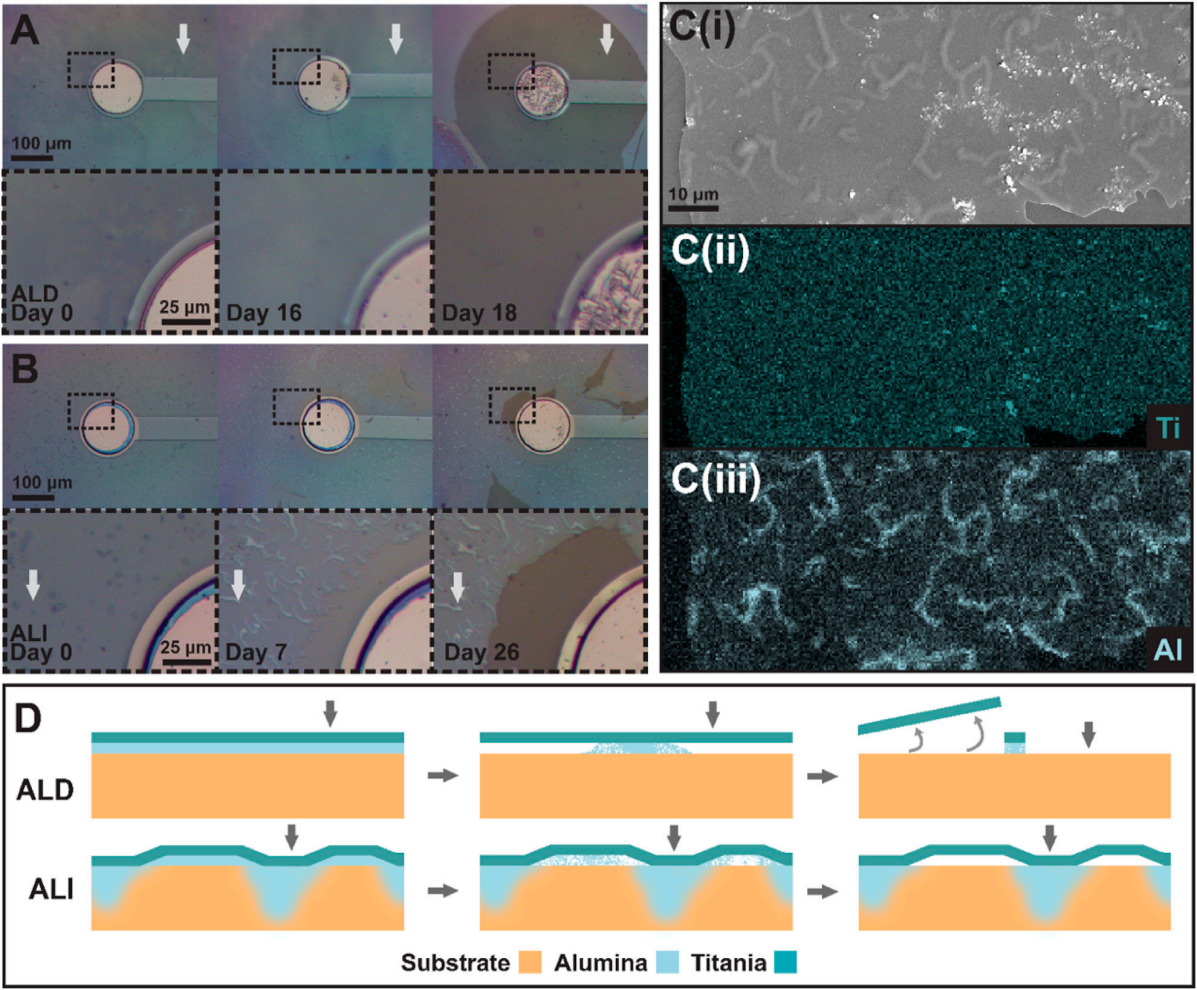
Progression of coating degradation near an electrode for **(A)** an ALD device and **(B)** an ALI device. Insets show the outlined region in greater detail. Note the presence of “squiggles” in **(B)**, which are not present in **(A)**. White arrows correspond to locations indicated by gray arrows in **(D)**. **(C)** (i-iii) show an SEM image of a degraded ALI coating and EDS maps of titanium and aluminum content, respectively, showing the concentration of aluminum in the “squiggles” visible in the SEM micrograph in contrast to the uniform distribution of titanium. **(D)** illustrates the ALI anchoring theory, with failure progression of an ALD coating shown in the top row, and of an ALI coating in the bottom row. From left to right, devices are depicted as-fabricated, in the midst of alumina dissolution, and after most alumina dissolution has occurred. Gray arrows correspond to the locations indicated by white arrows in **(A)** for the ALD device and **(B)** for the ALI device.

While coating strategy (ALI, ALD, or uncoated control) did not appear to have a quantitative effect on device lifetime, we observed interesting qualitative differences between the progression of failure in ALI and ALD coatings, illustrated in Figures 9, 10. It was predicted that based on the vulnerable areas at the device sidewall and electrode vias, coating failure for ALD devices would naturally initiate at and progress from those areas, whereas this effect should be reduced for ALI devices on which those areas are protected. Indeed, this trend was generally observed in practice. Figure 9 shows optical micrographs of the electrode sites and substrate edges of two devices, one with an ALD coating (Figure 9, top) and one with an ALI coating (Figure 9, bottom). By day 16, the area immediately surrounding some of the electrode sites of the ALD device had already lost its coating, whereas the coating is intact on all of the ALI sites. By day 26, nearly all of the coating had been lost from the area surrounding the ALD device’s electrodes, but was largely intact around those of the ALI device. Similarly, by day 10, coating loss was visible along the edge of the ALD device, but not the ALI device. By day 21, most of the coating on the ALD device’s edge was gone, while the ALI device’s was still present.

In general, we found that due to the presence of alumina on the bottom of both coatings, the metal oxide layer would gradually be undercut as the alumina dissolved in the presence of saline. However, the way in which the dissolution progressed differed between ALD and ALI devices. Figure 10 shows two sets of electrode sites, from an ALD device (Figure 10A) and an ALI device (Figure 10B). Both coatings are completely intact on day 0. By day 16, though, a uniform ring or bubble of discoloration is visible upon close inspection around the electrode site of the ALD device, where the alumina has been undercut. Two days later, on day 18, the entire undercut area lifted off at once. This is consistent with dissolution beginning at the circular electrode site and progressing in a uniform manner in all directions. On the ALI device, however, the behavior is different. While some discoloration suggestive of alumina dissolution is present around the electrode by day 7, the coating is still largely intact (or at least present) many days later, on day 26. It was observed that ALI devices tended to develop visible “squiggles” in their coatings over time, but their origin, nature, and effect on failure progression were unclear. SEM/EDS was performed on a failed device to probe the squiggles’ characteristics (Figure 10C(i-iii)). Per EDS, consistent with the alumina undercut theory, the titania layer was still present in a totally uniform layer wherever the oxide coating remained. However, the aluminum signal was no longer uniformly distributed; instead, aluminum appeared to be concentrated in the areas where “squiggles” were present. The reason for this is still not well understood, but given that the progression of alumina dissolution and coating loss appeared to be interrupted in areas where squiggles were present, it appeared that the squiggles served to anchor the titania layer to the polymer substrate in some way. A possible explanation for the squiggles could be nonuniform infiltration, as referenced earlier. Areas in which the alumina experienced greater hybridization with the polymer substrate might experience dissolution at a slower rate due to lower accessibility of the alumina to the solvent, rather than the completely unimpeded progress seen in the ALD coating. This concept is illustrated in Figure 10D.

## 4 Discussion

Overall, as shown, the 3D-ALI strategy did appear to qualitatively improve the failure behavior of the encapsulation, both by reducing failure initiation at vulnerable sidewalls and by interrupting the dissolution of alumina and subsequent flaking off of the metal oxide layer, but further improvements related to stress-related failure and materials selection are required. It is important to note that the edge-initiated failure seen here in ALD coatings is likely to be more detrimental in final-design therapeutic devices due to their greater miniaturization compared to our test devices. Our devices had large distances >1 mm between the device sidewall and the first metal trace, as well as between electrode sites. On fully miniaturized devices with high channel counts and minimized dimensions, electrodes will be much closer to each other, and metal traces may be much closer to device sidewalls. Therefore, any loss of ALD coating initiating and progressing from these vulnerable areas will reach important areas sooner, and may be more likely to coalesce into even larger failed regions as dissolution fronts reach each other. Therefore, the relative advantages of ALI coatings may be more apparent in these situations. Another such advantage of the 3D-ALI encapsulation was its reduced susceptibility to bending-induced cracking as compared to the conventional ALD encapsulation. This improved response to mechanical stresses should extend to repeated/cyclical loading, but this was not examined in the current study. Additional tests involving cyclical loading should be incorporated in the future in order to better reflect the loading conditions experienced by implanted devices.

It is worth questioning why, in the case that the ALI coatings failed more slowly, they did not quantitatively improve the lifespan of the electrodes as compared to ALD devices. The most likely explanation may simply be that the most relevant failure modes were not driven by moisture permeation through the top and bottom polyimide, which is what the encapsulation is intended to reduce. Most failures that were not related to metal cracking appeared to initiate at the electrode site and travel along the trace from there, even when the electrode via sidewall was protected by ALI, thus somewhat bypassing the protective effects of the encapsulation. This was likely possible because on the surface of the electrode where infiltration is not possible, ALD and ALI were equivalent, and so there is still an accessible point for alumina dissolution to begin. From there, the dissolution may spread up the sidewall and out onto the device. The benefits of an alternative non-alumina base layer for the metal oxide coating are obvious, but candidates for such are limited, due to the characteristics of the precursors available for less-soluble oxides such as hafnia and titania. The molecular size of their precursors is often much larger, as the metal atom may be contained in a tetrakis (dimethylamino) or isopropoxide rather than trimethyl compound, the larger sizes of which hinder the effectiveness of infiltration (Ren et al., 2021). Physically smaller precursors such as titanium tetrachloride (TiCl_4_) exist but have their own considerations; in the case of TiCl_4_, the formation of highly corrosive hydrochloric acid as a reaction byproduct necessitates the use of specialized vacuum pump and line equipment not easily accessible for all researchers (Chung et al., 2021). Including a further conformal polymer coating on top of the metal oxide bilayer such as Parylene C could offer additional lifetime improvement, but would not solve the underlying issue of alumina dissolution (Xie et al., 2014). There were also some indications of platinum dissolution over time based on the appearance of anodic CV peaks. If this is the case, reducing the cathodic CV potential to −0.3V vs. Ag/AgCl or adding a compatibilizing coating such as PEDOT:PSS could help minimize this effect in the future (Shah et al., 2024, p. 202; Dijk et al., 2021). Another way to improve device lifetimes by way of reducing PI-trace delamination could be the addition of an adhesion promoter such as APTES as mentioned earlier. An adhesion promoter was not included in this study because of the potentially sensitive nature of the partially-cured PI base layer; it was uncertain how the addition of an adhesion promoter would impact the adhesion benefits imparted by the partial cure. Additional experiments assessing interlayer adhesion strength via peel test could clarify whether an adhesion promoter can be implemented to improve PI-metal adhesion without affecting the good PI-PI adhesion attained from a partially cured base layer.

Additionally, the mean times-to-failure reported here are not particularly impressive, and are roughly on par with or slightly below what one would expect based on similar studies (Mariello et al., 2022; Guljakow and Lang, 2023). One possible factor in this may have been the choice of 87°C as an aging temperature: while a high acceleration factor is a necessity for devices intended to have lifetimes of many years, polyimide has been shown to experience failure modes at such elevated temperatures which do not appear at lower temperatures (for example, PI mechanical properties did not degrade over time at 60°C, but did at 85°C (Rubehn and Stieglitz, 2010)), making extrapolations of lifetime between testing temperatures difficult. Future studies may need to accept a lower acceleration factor and correspondingly longer study durations, or otherwise implement additional means of accelerating aging beyond temperature, such as reactive accelerated aging (Gong et al., 2020; Manatchinapisit et al., 2022). Finally, an interesting future study could perhaps be performed analyzing the differences in failure modes between the 3D-ALI strategy, which encapsulates the outside of the device, and the direct encapsulation of metal tracks on the interior of the device such as that employed by Duran-Martinez et al. (2021). The direct metal encapsulation strategy could provide better protection against metal corrosion but might not protect against adhesion failure between top and bottom polymer layers, for example. It also may be possible to combine these approaches to achieve both types of enhancement.

## 5 Conclusion

Although the infiltrated coating did not appear to significantly extend device lifespans as desired, the study successfully achieved its other objectives: developing a method to encapsulate entire active electrode devices with an infiltrated metal oxide layer, and demonstrating that this coating reduces failure initiation from the vulnerable areas it was designed to protect. Further optimization is needed to fully realize the benefits of this coating strategy, such as reducing or eliminating metal cracking through the use of serpentine tracks and flexible connectors, or by replacing the alumina-infiltrated layer with a more dissolution-resistant material. As more devices using next-generation encapsulations are implemented on flexible substrates, the design considerations described herein—mitigation of sidewall vulnerabilities and substrate-encapsulation mechanical compatibility—will continue to be important factors, and creative solutions such as our combination of on- and off-wafer processes will be needed. Though more work remains to be done, through these developments and others, maximally optimized devices capable of sustaining benefits to patients for the necessary timescales become one step closer to practical implementation and widespread usage.

## Data Availability

The raw data supporting the conclusions of this article will be made available by the authors, without undue reservation.
