# Supplementary material for: Failure behavior of polymer microelectrode arrays encapsulated with conventional ALD and 3D-ALI barriers
Source: Front Bioeng Biotechnol. 2025 Jul 24;13:1622927. doi: 10.3389/fbioe.2025.1622927 (PMC12328438; doi:10.3389/fbioe.2025.1622927)
Supplement: Supplementary file 1 [file DataSheet1.pdf]

Supplementary Information

Failure behavior of polymer microelectrode arrays encapsulated with conventional ALD and 3D-ALI barriers

Martin Niemiec[1], Necmi Biyikli[2,3], Fatih Bayansal[2,3], Kyungjin Kim[1,3,4*]

[1] Department of Biomedical Engineering, University of Connecticut, Storrs, Connecticut, USA
[2] Department of Electrical and Computer Engineering, University of Connecticut, Storrs, Connecticut, USA
[3] Institute of Materials Science, University of Connecticut, Storrs, Connecticut, USA
[4] Department of Mechanical Engineering, University of Connecticut, Storrs, Connecticut, USA

*** Correspondence:**
Kyungjin Kim
kyungjin.kim@uconn.edu

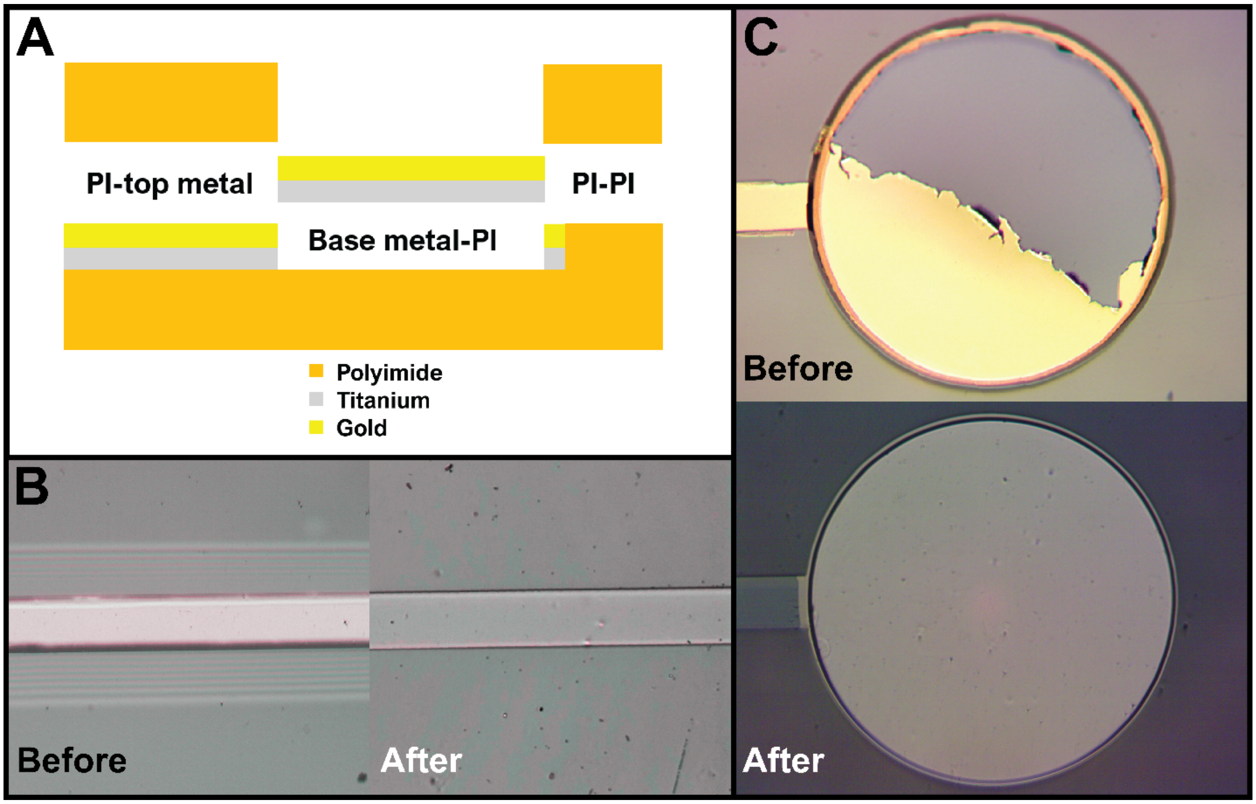

Figure S1: Adhesion improvements facilitating damage-free sonication. (A) Schematic showing interlayer adhesions which affect process success, including polyimide:top metal, polyimide:bottom metal, and polyimide:polyimide. (B) Before and after of improvements to polyimide:top metal adhesion, showing elimination of polyimide:top metal delamination. (C) Before and after of improvements to polyimide:bottom metal showing elimination of electrode damage.

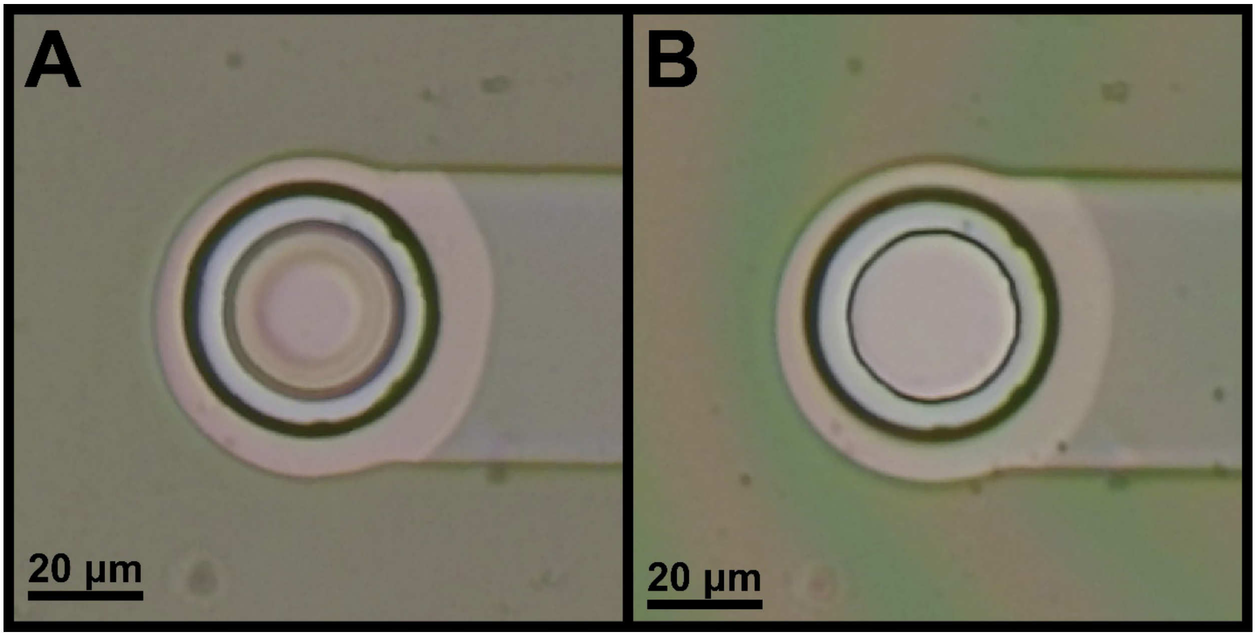

Figure S2: Before (A) and after (B) comparison of sonication-assisted encapsulation via opening. ALI/ALD-coated photoresist is visible as a reddish circle before via opening, but is no longer present afterward, with only a ring of ALI/ALD material remaining around the outside of the electrode where no resist was present.

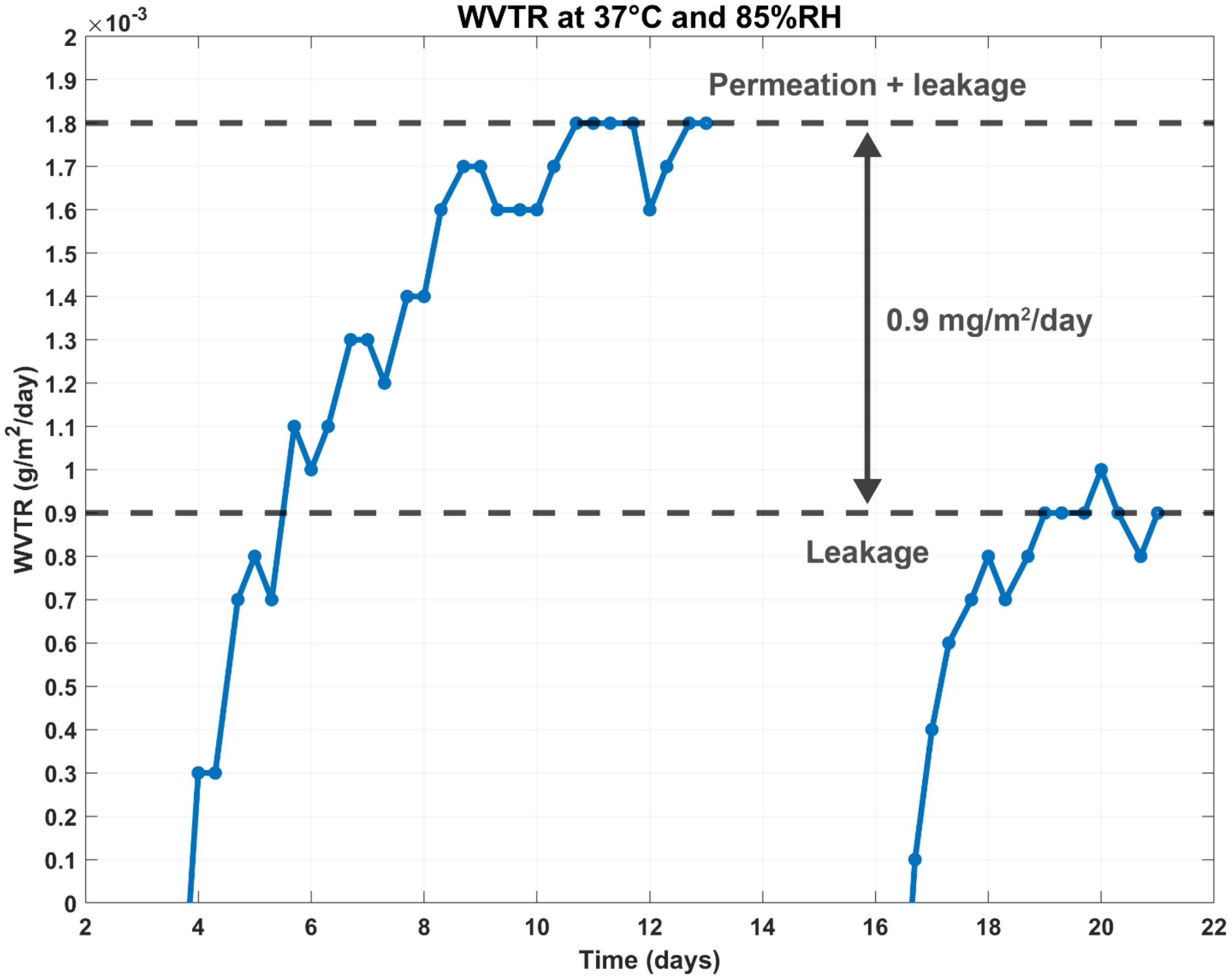

Figure S3: Full water vapor permeation rate (WVTR) test of 100 cycles alumina ALI + 250 cycles ALD titania on 5 mil Kapton® HN film. First blue trace shows WVTR measured when generated humidity was set to 85% (permeation + leakage); second blue trace shows WVTR measured when humidity was set to 0% (external leakage only). The difference between the two is the corrected WVTR of the film.

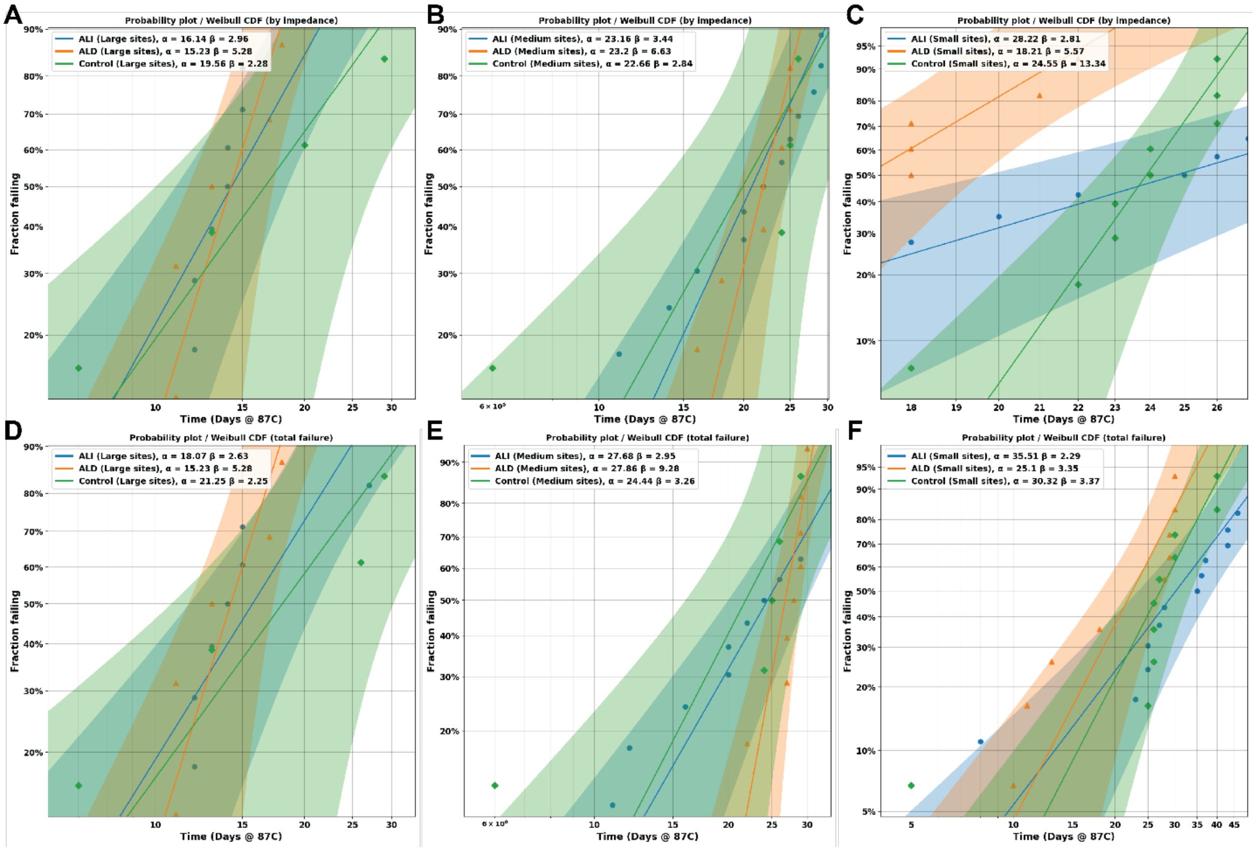

Figure S4: Additional Weibull CDF plots. (A), (B), and (C) show Weibull CDFs for large (100kμm^2), medium (10kμm^2), and small (1kμm^2) sites, with ALI data shown in blue, ALD in orange, and uncoated controls in green, when analyzing by impedance deviation (failure occurs when impedance varies by more than (+/-) 50%). Shaded areas represent 95% confidence intervals for each fit. (D), (E), and (F) show the same when analyzing by total failure instead.

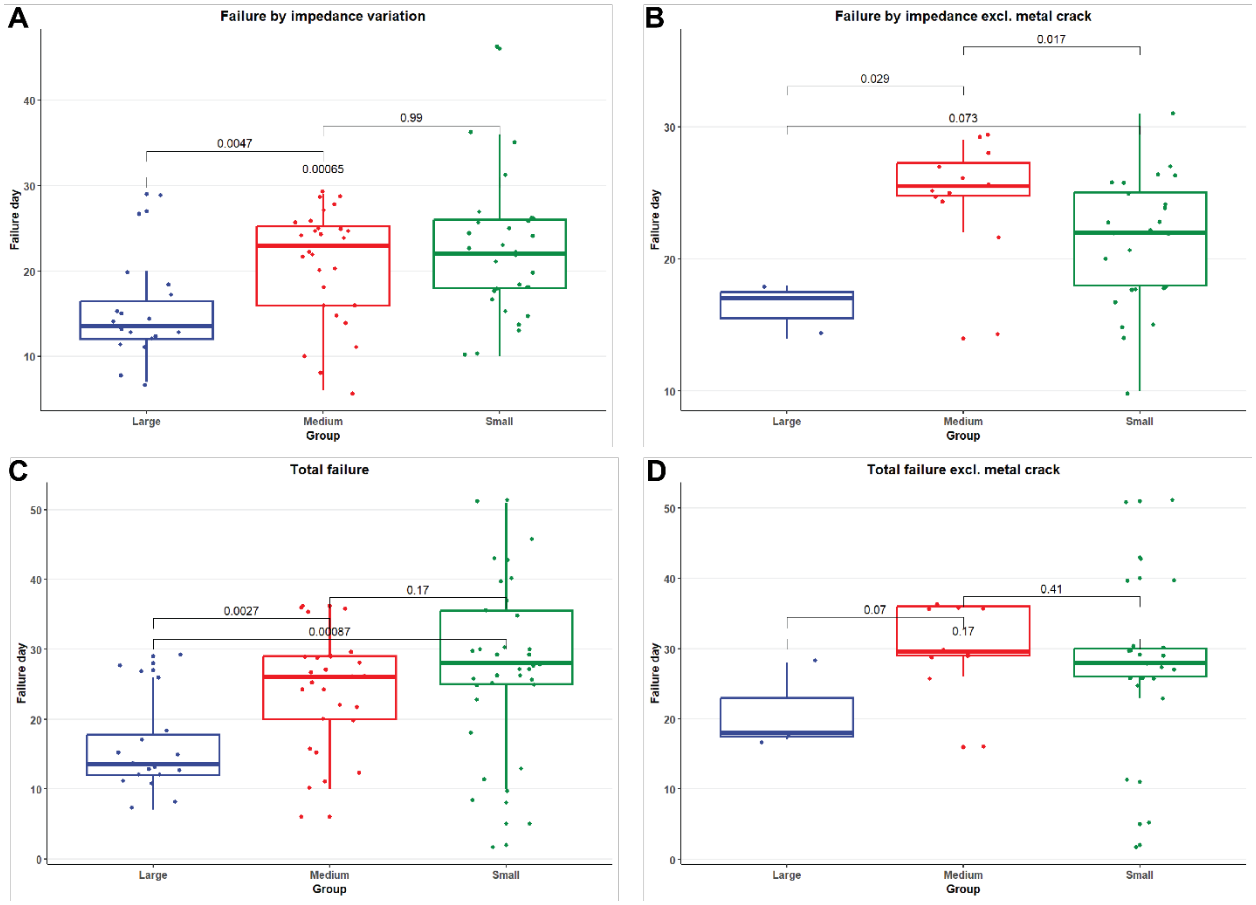

Figure S5: Box plots of failure based on electrode size with and without metal cracking. Interquartile range is given by the top and bottom edges of the box, with the horizontal line indicating the median value. Significances as determined by Wilcoxon rank-sum test are shown above the boxes. (A) and (B) define failure by impedance deviation of (+/-) 50% from stable value, with (B) excluding failure due to metal cracking. (C) and (D) show the same when analyzing by total failure. In both cases, the difference between large sites and the other sizes becomes non-significant when metal crack failures are excluded.
